# The Mechanism of Markovnikov-Selective Epoxide Hydrogenolysis Catalyzed by Ruthenium PNN and PNP Pincer Complexes

**DOI:** 10.1021/acs.organomet.2c00503

**Published:** 2023-02-27

**Authors:** Marianna C. Head, Benjamin T. Joseph, Jason M. Keith, Anthony R. Chianese

**Affiliations:** Department of Chemistry, Colgate University, 13 Oak Drive, Hamilton, New York 13346, United States

## Abstract

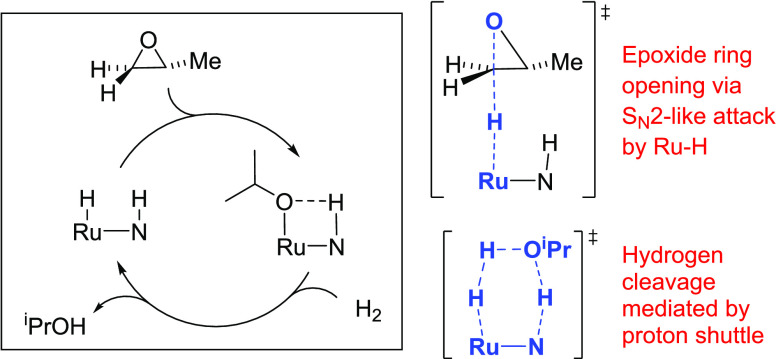

The homogeneous catalysis of epoxide hydrogenolysis to give alcohols has recently received significant attention. Catalyst systems have been developed for the selective formation of either the Markovnikov (branched) or anti-Markovnikov (linear) alcohol product. Thus far, the reported catalysts exhibiting Markovnikov selectivity all feature the potential for Noyori/Shvo-type bifunctional catalysis, with either a RuH/NH or FeH/OH core structure. The proposed mechanisms of epoxide ring-opening have involved cooperative C–O bond hydrogenolysis involving the metal hydride and the acidic pendant group on the ligand, in analogy to the well-documented mechanism of polar double-bond hydrogenation exhibited by catalysts of this type. In this work, we present a combined computational/experimental study of the mechanism of epoxide hydrogenolysis catalyzed by Noyori-type PNP and PNN complexes of ruthenium. We find that, at least for these ruthenium systems, the previously proposed bifunctional pathway for epoxide ring-opening is energetically inaccessible; instead, the ring-opening proceeds through opposite-side nucleophilic attack of the ruthenium hydride on the epoxide carbon, without the involvement of the ligand N–H group. For both catalyst systems, the rate law and overall barrier predicted by density functional theory (DFT) are consistent with the results from kinetic studies.

## Introduction

Homogeneous catalysts for the hydrogenation of polar compounds provide a selective, atom-economical alternative to traditional approaches based on stoichiometric hydride donors such as LiAlH_4_ and NaBH_4_. Ruthenium catalysts developed by Noyori^1^ and Shvo^2^ are highly effective for substrates such as ketones and imines, and the scope of accessible substrates was greatly expanded following the discovery by Milstein and co-workers of a pincer–ruthenium catalyst effective for the hydrogenation of esters.^3^ Following this report, methods were developed for the hydrogenation of an increasing range of polar substrates including amides,^4^ carbonate esters,^5^ urea derivatives,^6^ and carbon dioxide.^7^ The most efficient catalysts frequently contain a ligand N–H group in close proximity to the metal center,^8^ which mechanistic studies have shown has a key role in the bifunctional activation of dihydrogen and its transfer to polar substrates.^8c,8f,8h,9^ In some cases where the originally developed ruthenium precatalyst lacks such an N–H group, subsequent work showed that the precatalyst transforms to an N–H-containing “Noyori-type” form under the conditions of catalysis.^8i,10^

Although most research in this area has focused on the hydrogenation of substrates containing C–O or C–N double bonds, recent work has shown that epoxides are reactive under similar conditions, undergoing hydrogenolysis of a C–O single bond to give alcohols with regioselectivity dependent on the choice of catalyst. Some catalyst systems selectively promote formation of the anti-Markovnikov (linear) product. For example, Yao et al. reported the anti-Markovnikov-selective hydrogenolysis of epoxides catalyzed by a Ti–Co dual catalytic system and proposed a radical-based mechanism for hydrogen activation and transfer.^11^ Scheuermann and co-workers reported that an iridium(I)–pincer complex in combination with triflic acid catalyzes anti-Markovnikov-selective epoxide hydrogenolysis^12^ and later demonstrated that acid-catalyzed epoxide hydrolysis gives the 1,2-diol, whose hydrogenation to the terminal alcohol is catalyzed by iridium nanoparticles generated in situ.^13^ Several anti-Markovnikov-selective systems employing a combination of transition-metal catalysis and a Brønsted or Lewis acid were shown to proceed initially through acid-catalyzed Meinwald rearrangement of the epoxide to the aldehyde, followed by transition-metal-catalyzed hydrogenation of the aldehyde. These include an Fe/tetraphos/trifluoroacetic acid system^14^ and a Co/triphos/Zn(OTf)_2_ system^15^ reported by Beller, as well as an erbium–cobalt dual system reported by Werner.^16^

**Figure 1:**
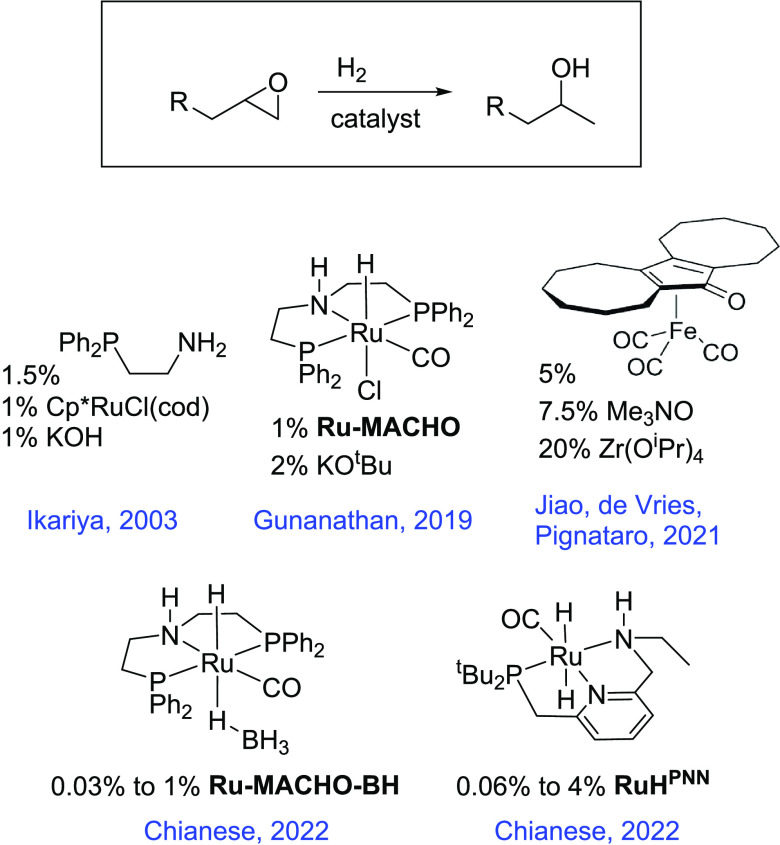
Reports of Markovnikov-selective epoxide hydrogenation.

On the other hand, some catalyst systems selectively give the Markovnikov (branched) alcohol products. Figure 1 summarizes these catalyst systems, all of which share the potential for Noyori-type bifunctional catalysis through the synergistic action of a Lewis-acidic metal center and a pendant N–H or O–H group. Ikariya reported that Cp*RuCl(1,5-cyclooctadiene) in combination with Ph_2_PCH_2_CH_2_NH_2_ and KOH catalyzes the Markovnikov-selective hydrogenolysis of epoxides and proposed a concerted, Ru–H/N–H bifunctional mechanism for hydrogen transfer to the epoxide, analogous to Noyori’s mechanism for the addition to C=O double bonds.^17^ Gunanathan reported that the PNP pincer complex **Ru-MACHO** in combination with KO*^t^*Bu catalyzes Markovnikov-selective epoxide hydrogenolysis and proposed the same mechanism.^18^

In an important finding, Jiao, de Vries, Pignataro, and co-workers recently reported that a Knölker-type iron cyclopentadienone complex catalyzes epoxide hydrogenolysis with regioselectivity dependent on the Lewis acid cocatalyst added and reported the first density functional theory (DFT) study on the reaction mechanism.^19^ These authors concluded that, in the absence of a Lewis acid cocatalyst, two different mechanisms have similar energy barriers and are competitive. One mechanism is analogous to the Noyori-type bifunctional mechanism proposed by Ikariya, featuring concerted transfer of hydride and proton from the reduced catalyst to the epoxide substrate. In the second mechanism, the ligand OH group on the hydrogenated catalyst intermediate promotes the Meinwald rearrangement of the epoxide to the aldehyde, which is subsequently hydrogenated following a Noyori-type mechanism. Using the model compound BF_3_ for computational studies, these authors showed that transition states for both the Meinwald rearrangement and the Noyori-type concerted hydrogen transfer are stabilized by the addition of a Lewis acid. Although addition of Al(OTf)_3_ as a cocatalyst promotes the formation of the linear product while Zr(O*^i^*Pr)_4_ favors the branched product, the origin of this regiocontrol was not determined. The authors employed the model Lewis acid BF_3_ in their computational work due to the difficulties in modeling the solution-state aggregation of the experimentally used Al(OTf)_3_ and Zr(O*^i^*Pr)_4_.

We recently reported that the ruthenium–pincer complexes **Ru-MACHO-BH** and **RuH**^**PNN**^ (Figure 1) are catalysts for the Markovnikov-selective hydrogenolysis of epoxides, giving turnover numbers up to 3000 without the need for additives such as strong bases or Lewis acids.^20^ Because these catalyst systems are highly active, feature strong-binding pincer ligands, operate with only one component, and are well-behaved kinetically (vide infra), they provide an ideal platform for a combined experimental and computational investigation of the mechanism. We report here that both catalysts operate through a pathway that has not been proposed in any of the prior reports; epoxide ring-opening occurs through an S_N_2-like opposite-side attack of Ru–H on the epoxide carbon, without the involvement of the ligand N–H group. The previously proposed Noyori-type pathway, which involves a same-side substitution of hydride for oxygen at the epoxide carbon, exists on the potential energy surface but has a prohibitively high energy barrier. For both catalysts, the overall energy barrier and rate law predicted through DFT are consistent with the kinetic data.

## Computational Mechanistic Analysis

### Background

**Scheme 1:**
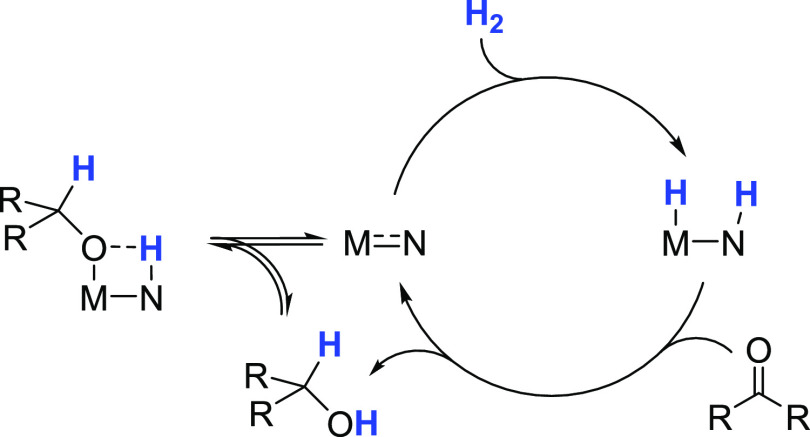
Mechanistic Overview of Noyori-Type Catalytic Hydrogenation

Due to the long history of development of bifunctional Noyori-type catalysts for the hydrogenation of polar substrates, there is a large body of computational work aimed at elucidating the mechanisms of these reactions.^8c,8f,8h,9a−9k,21^Scheme 1 shows a simplified overview of the accepted pathways for these reactions, which most commonly involve the hydrogenation of a C–O or C–N double bond; a ketone substrate is shown here. Hydrogen activation typically proceeds from an unsaturated metal–amide intermediate (center) and results in the heterolytic cleavage of the H–H bond, leaving a hydride on the metal center and a proton on the basic nitrogen (right). This hydrogen activation often proceeds with a lower barrier when an alcohol molecule (product or solvent) serves as a proton shuttle.^9f,22^ The metal hydride intermediate then reduces the substrate, transferring the hydride to the carbon atom and the proton to the oxygen atom.^9f,9l,23^ Substrate reduction may also benefit from the inclusion of an explicit alcohol molecule.^9l,23b^

Metal alkoxide intermediates (left) can play an important role in catalysis. One possibility, shown in Scheme 1, is that a metal alkoxide formed by addition of the O–H bond across the M–N bond serves as an off-cycle intermediate, potentially resulting in product inhibition.^23a^ Alternatively, the metal alkoxide can be an on-cycle intermediate; several studies have found minimum-energy pathways (MEPs) or competitive pathways that cycle between an alkoxide intermediate and a metal-hydride intermediate.^9l,23^ In some cases, the N–H group on the ligand can play a beneficial role as a hydrogen-bond donor without being deprotonated.^23b,24^ In determining the detailed mechanism for a particular catalytic reaction, it is essential to explore thoroughly the possible pathways for hydrogen activation and substrate reduction and equally important to search for stable intermediates. It is possible that an alkoxide intermediate, an unsaturated intermediate, a hydride intermediate, or even a more exotic adduct^25^ can serve as the resting state, which, along with the highest-energy transition state, determines the catalytic rate.

Because, as described below, the rate laws differ for epoxide hydrogenolysis catalyzed by the **RuPNP** and **RuPNN** systems, we have thoroughly analyzed both catalysts through density functional theory. We find that the two catalysts operate by closely analogous mechanisms but display different kinetics, arising from different resting-state speciation.

### Minimum-Energy Pathway (MEP) for Epoxide Hydrogenolysis

Throughout this work, we refer to the catalyst derived from **Ru-MACHO-BH** as the **RuPNP** system and the catalyst derived from **RuH**^**PNN**^ as the **RuPNN** system. Because most structures are analogous in the two pathways, we have used a common set of names for intermediates and transition states and distinguish the two catalyst systems with an appended **PNP** or **PNN** suffix as appropriate. In the computational work, we have used complete, nontruncated catalyst structures, and we chose the simple epoxide substrate propene oxide to minimize complications arising from multiple substrate conformations. In the kinetic study described later, we used the substrate 1-tetradecene oxide, which minimizes evaporation and facilitates accurate quantitation, and is expected to be sterically and electronically similar to propene oxide.

**Figure 2:**
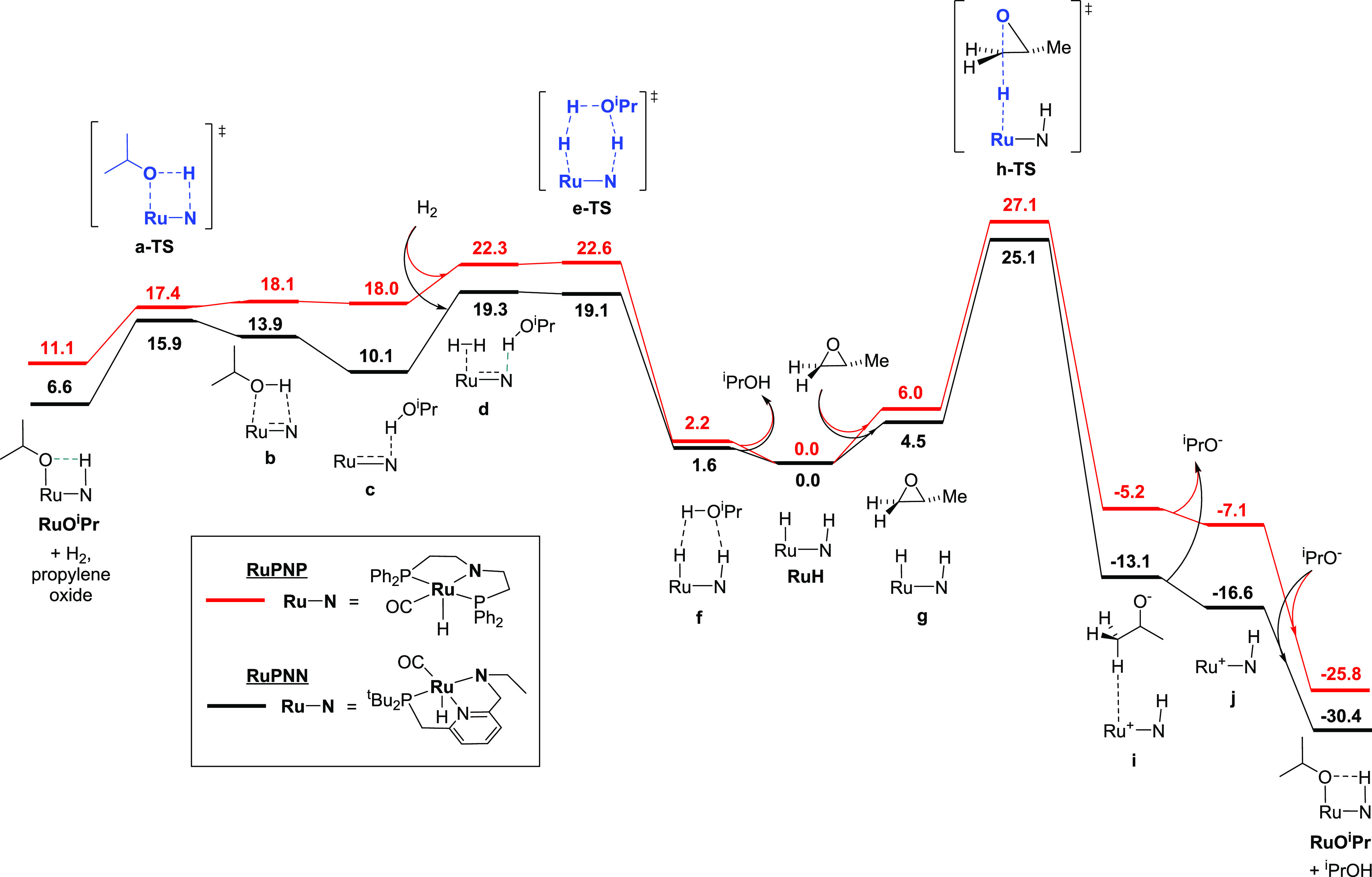
MEPs for the **RuPNP**- and **RuPNN**-catalyzed hydrogenation of propylene oxide. Throughout this work, atoms in bold and blue represent the atoms principally involved in bond-breaking and bond-forming events in transition states. Energies given represent free energies in kcal/mol at 356.15 K (83 °C), corrected to 1.0 M concentrations in solution, relative to **RuH** and the small-molecule reactants.

Figure 2 shows the complete MEPs for the hydrogenolysis of propylene oxide catalyzed by the **RuPNP** (red) and **RuPNN** (black) systems. The two catalysts operate via analogous pathways, with similar energetics. The sequence for hydrogen activation begins with the alkoxide **RuO***^**i**^***Pr** and proceeds to the dihydride **RuH** through a precedented pathway.^9l,23c^ First, the alkoxide oxygen deprotonates the pincer ligand nitrogen through **a-TS** to give the coordinated alcohol species **b**. We note that for the **RuPNN** system, convergence of **a-TS** in continuum solvent was unsuccessful despite multiple attempts. This species was optimized in the gas phase, and the gas-phase free-energy correction was applied to the single-point electronic energy calculated using a continuum solvent. Then, the alcohol oxygen dissociates from ruthenium to give the unsaturated intermediate **c**. Next, dihydrogen enters and forms the σ-complex **d**. The H–H bond is cleaved through the proton-shuttle transition state **e-TS**, giving first the alcohol-stabilized dihydride **f**, which releases 2-propanol to give the dihydride resting state **RuH**. In developing the MEPs, we conducted a search for plausible catalyst resting states, which is described in the Supporting Information (SI). As part of this search, we assessed the effect of including vs excluding an explicit, hydrogen-bonded solvent (2-propanol) molecule. As shown in Figure S1, we find a minimal effect of removing the hydrogen-bonded solvent molecule for intermediate **c**. In contrast, we located transition states for H–H bond cleavage without a 2-propanol molecule included as a proton shuttle and found the free energies to be significantly higher at 32.3 kcal/mol for the **RuPNP** system and 28.8 kcal/mol for the **RuPNN** system (see the SI for details). The MEPs shown in Figure 2 include the solvated intermediates **c** and **d**, as explained in p S2.

The dihydride species **RuH**, defined as the free-energy reference in this diagram, is predicted to be the catalyst resting state and turnover-frequency-determining intermediate (TDI), as described below in the section titled “Predicted Kinetics.” Species **RuH** forms dispersion complex **g** with the epoxide substrate. The hydride then attacks the terminal epoxide carbon to give ion pair **i** through the S_N_2-like transition state **h-TS**. Ion pair **i** rearranges to alkoxide **RuO***^**i**^***Pr** through the completely dissociated ions **j** and 2-propoxide, completing the catalytic cycle. Although it may be possible to locate a nondissociative pathway for the rearrangement of **i** to **RuO***^**i**^***Pr**, we did not search for such a pathway because the dissociation into free ions is calculated to be exergonic in 2-propanol solvent. For the **RuPNN** system, we considered the addiction of an explicit 2-propanol solvent molecule to **h-TS**, expecting that it might be necessary to stabilize the developing negative charge on oxygen. This pathway, shown in the Supporting Information, had a higher barrier of 31.0 kcal/mol.

### Regioselectivity

**Figure 3:**
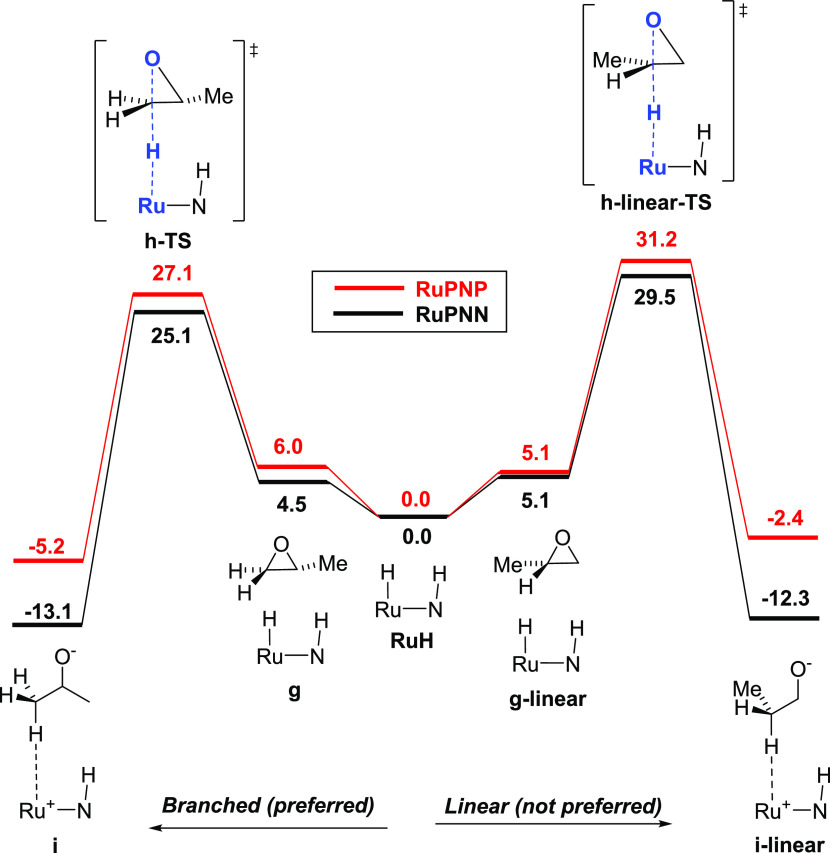
Comparison of the branched (left) and linear (right) pathways for epoxide ring-opening. For both the **RuPNP** and **RuPNN** systems, the branched pathway is preferred by approximately 4 kcal/mol.

Both the **RuPNP** and **RuPNN** systems are selective for the formation of the branched, Markovnikov product of epoxide hydrogenolysis, giving less than 1% of the linear, anti-Markovnikov product for aliphatic substrates such as 1-tetradecene oxide.^20^ For both systems, we located transition states **h-linear-TS** that lead to the linear 1-propanol product and found them to be higher in free energy than the corresponding transition states **h-TS** leading to branched products by 4.1 (**RuPNP**) and 4.5 kcal/mol (**RuPNN**) (Figure 3). These energy differences are consistent with the high branched selectivity of these catalysts and can be rationalized through steric effects. In the branched pathways (Figure 3, left), the ruthenium hydride attacks the terminal, primary carbon of the epoxide, while the formation of the linear product (Figure 3, right) proceeds through hydride attack on the internal, secondary carbon of the epoxide.

### Alternative, Noyori-Type Pathway for Epoxide Ring-Opening

**Figure 4:**
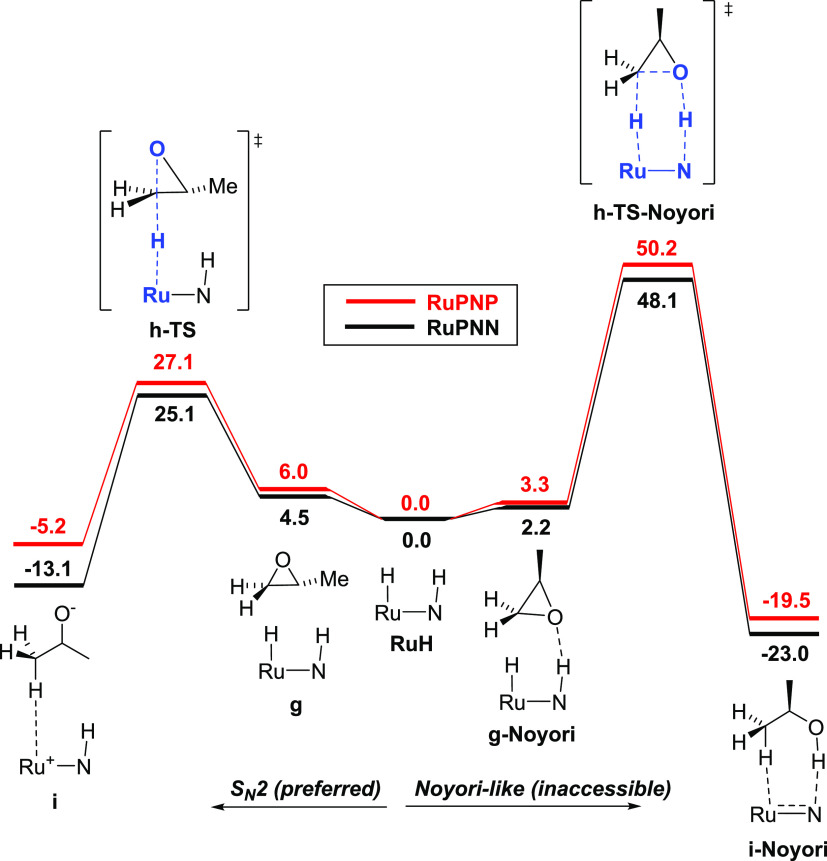
Comparison of S_N_2 (left) and Noyori-like (right) transition states for epoxide ring-opening. For both **RuPNP** and **RuPNN** systems, the S_N_2 pathway is preferred by approximately 23 kcal/mol.

In our MEPs for both the **RuPNP** and **RuPNN** systems, the epoxide ring-opening proceeds through the transition state **h-TS**, representing an S_N_2-like opposite-side attack of the ruthenium hydride on the terminal carbon of the epoxide (Figure 4, left). In these transition states, the epoxide oxygen with its developing negative charge points away from the ruthenium complex, and the N–H group of the pincer ligand is not involved in a hydrogen bond. This is in direct contrast with the proposals by Ikariya^17^ and Gunanathan^18^ and the computed pathway of Jiao, de Vries, Pignataro, and co-workers,^19^ all of which involve a same-side attack of hydride on the epoxide carbon concomitant with C–O cleavage and proton transfer from the ligand N–H (or O–H) to the epoxide oxygen. We were able to locate the transition states **h-TS–Noyori** corresponding to this same-side substitution for the **RuPNP** and **RuPNN** systems (Figure 4, right), and we find them to be energetically inaccessible, each approximately 23 kcal/mol above the S_N_2-like transition states.

### Predicted Kinetics

With the complete MEPs for epoxide hydrogenolysis catalyzed by the **RuPNP** and **RuPNN** systems determined, it is possible to predict the rate laws for these reactions, for comparison with experimental kinetic data. The energetic span model offers a straightforward method to predict the rate law in cases with a clearly defined turnover-frequency-determining intermediate (TDI, equivalent to the resting state) and turnover-frequency-determining transition state (TDTS).^26^ In essence, only the species that enter or leave the sequence after the TDI and before the TDTS are included in the rate law.

**Scheme 2:**
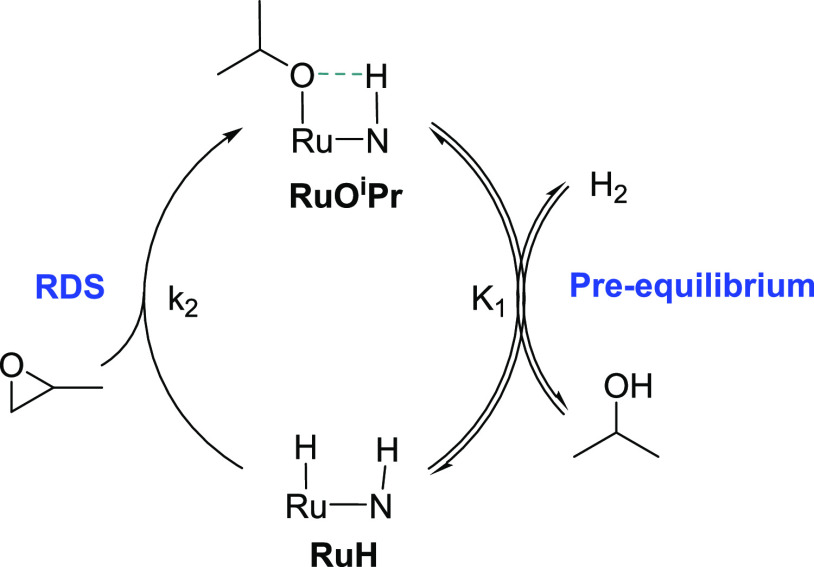
Simplified Scheme Determining the Kinetics for Epoxide Hydrogenolysis for the **RuPNN** System

The **RuPNP** catalyst is an example of a clearly defined system, with only the dihydride **RuH**^**PNP**^ as TDI and the epoxide ring-opening transition state **h-TS**^**PNP**^ as TDTS. Since one molecule of epoxide enters the sequence between **RuH**^**PNP**^ and **h-TS**^**PNP**^, the rate is expected to depend on [epoxide] in a first-order manner. This gives the simple rate law shown in eq 1. Since dihydrogen enters the sequence before intermediate **RuH**^**PNP**^, the hydrogen pressure is not expected to influence the reaction rate. The product alcohol also departs the sequence before intermediate **RuH**^**PNP**^, so it is not expected to inhibit the reaction. The energetic span from **RuH**^**PNP**^ to **h-TS**^**PNP**^, which determines the overall rate of the catalytic reaction, is predicted to be 27.1 kcal/mol.
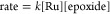
1The **RuPNN** system follows a closely analogous pathway to the **RuPNP** system, but the lower standard-state free energy of the alkoxide intermediate **RuO***^**i**^***Pr**^**PNN**^ raises the possibility that both **RuO***^**i**^***Pr**^**PNN**^ and **RuH**^**PNN**^ may be partially involved in determining the turnover frequency, as discussed in detail in the Supporting Information. Scheme 2 outlines the scenario: **RuO***^**i**^***Pr**^**PNN**^ and **RuH**^**PNN**^ are expected to establish a rapid pre-equilibrium, followed by rate-determining product formation from **RuH**^**PNN**^ through **h-TS**^**PNN**^. If **RuO***^**i**^***Pr**^**PNN**^ predominates substantially over **RuH**^**PNN**^ at the steady state, the reaction will display first-order kinetics in both hydrogen and epoxide. If **RuH**^**PNN**^ predominates substantially over **RuO***^**i**^***Pr**^**PNN**^, the reaction will be zero-order in hydrogen and first-order in epoxide, as is the case in the **RuPNP** system. If neither species predominates over the range of hydrogen concentrations studied, a nonlinear (saturation) dependence on hydrogen is expected. Following the steady-state approximation, the rate law shown in eq 2 is predicted (see the SI for the derivation). In the limiting case where the pre-equilibrium heavily favored **RuH**^**PNN**^, the energetic span would be 25.1 kcal/mol.
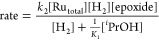
2

## Kinetic Experiments

**Scheme 3:**
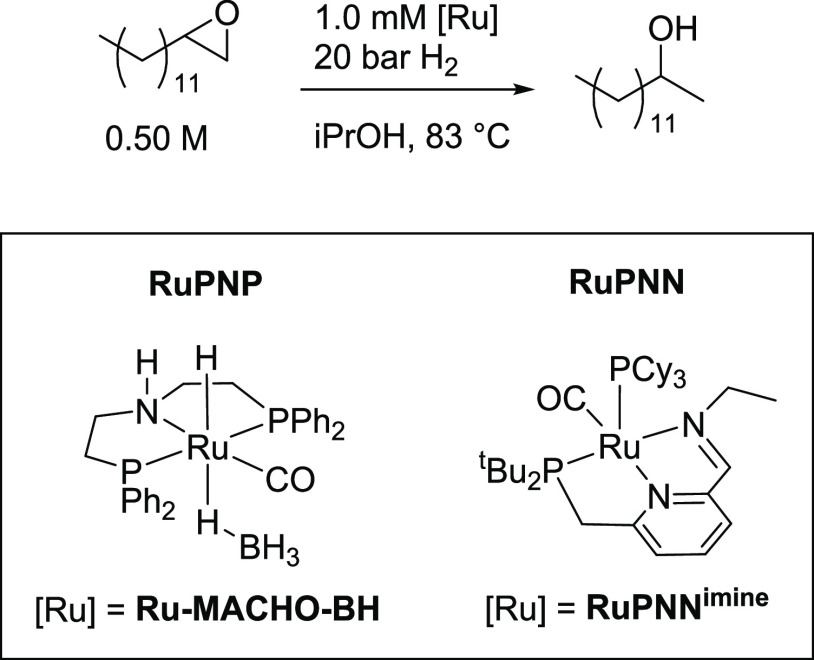
Standard Conditions for Kinetic Experiments

In the computational study described above, we used propylene oxide as the model epoxide, which allowed for the comparison of Markovnikov and anti-Markovnikov selectivity, while minimizing complications due to conformational flexibility. For kinetic analysis, we used 1-tetradecene oxide as the substrate, which is expected to be sterically very similar to propylene oxide. 1-Tetradecene oxide and the hydrogenolysis product 2-tetradecanol have low volatility, which minimizes evaporation during the reaction and after collecting aliquots. Additionally, this substrate simplifies kinetic modeling due to the selective formation of the Markovnikov product 2-tetradecanol under our experimental condition*s*. In previous work,^20^ we identified isopropyl alcohol as an ideal solvent for this reaction because of its high reaction rates and good selectivity for the Markovnikov product. Scheme 3 shows the standard reaction conditions. For the **RuPNP** system, we used **RuMACHO-BH** as a precatalyst, which serves as a base-free source of the dihydride **RuH**^**PNP**^.^7a,27^ For the **RuPNN** system, we used **RuPNN**^**imine**^ as a precatalyst, which is known to convert to **RuH**^**PNN**^ under hydrogen.^8i^ We conducted kinetic experiments for both the **RuPNN** and **RuPNP** systems, independently varying the initial concentrations of the substrate and catalyst, as well as the hydrogen pressure.

### RuPNP Kinetics

**Figure 5:**
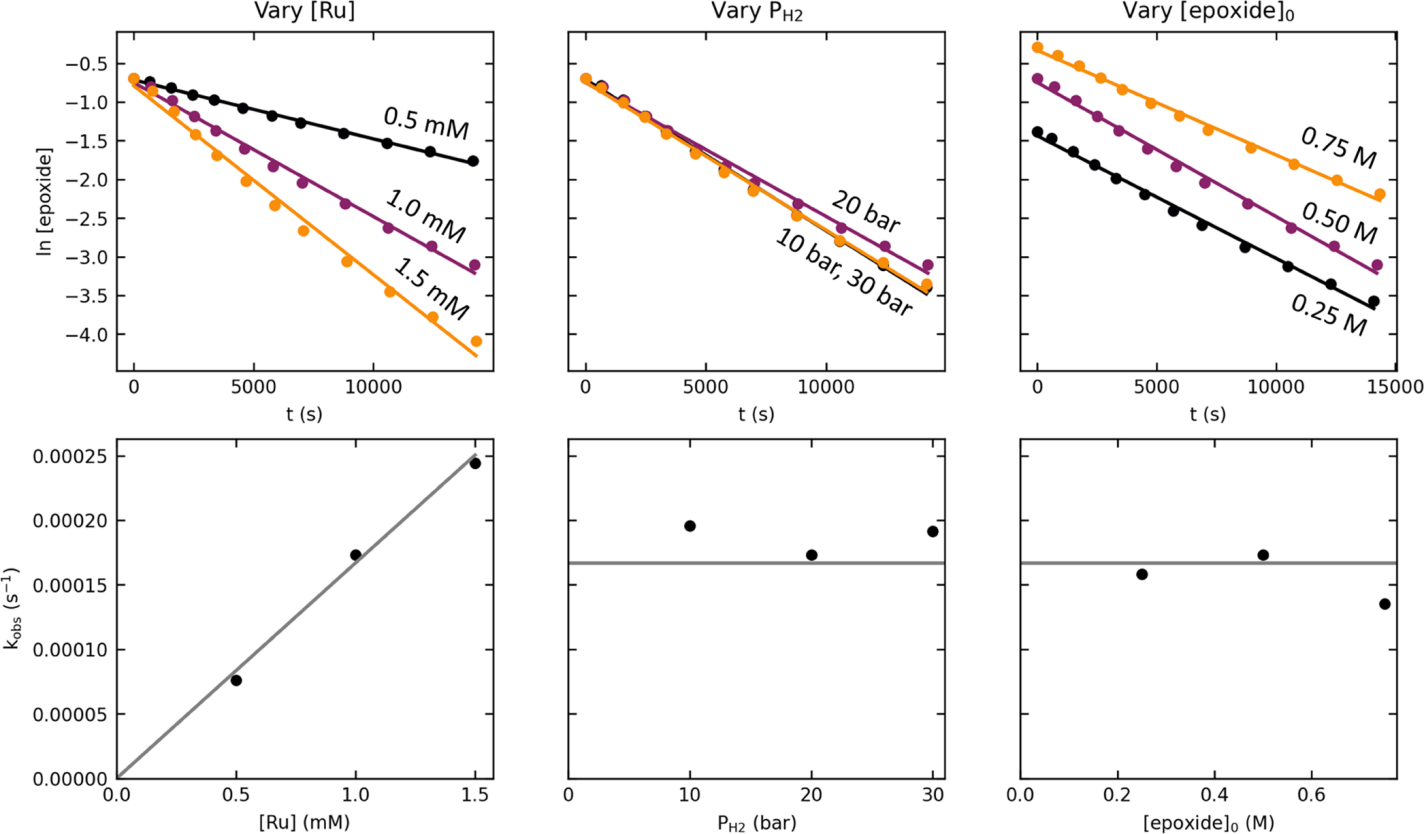
Kinetic data for 1-tetradecene oxide hydrogenolysis catalyzed by **RuMACHO-BH**. The top plots (points) show the time course of epoxide concentration using different initial concentrations of the Ru complex, hydrogen pressures, and initial concentrations of the epoxide. The solid lines represent linear fits to the natural logarithm of [epoxide] over time. The bottom plots show the *k*_obs_ values determined from the slopes of the linear fits (black points). The solid gray lines show the predicted *k*_obs_ values from a global fit of all seven experiments, according to the rate law shown in eq 1.

Figure 5 summarizes the kinetic data for the **RuPNP** system. We observed a linear relationship in the plot of ln[epoxide] vs time for all experiments, consistent with a first-order dependence of the rate on the epoxide concentration. The reaction showed a first-order dependence on [Ru] (left plots) and a zero-order dependence on the hydrogen pressure (middle plots). To probe for possible saturation in epoxide and product inhibition, we also conducted the reaction with varying initial concentrations of the epoxide (right plots). The minimal dependence of the rate on [epoxide]_0_ excludes these possibilities. We note that because the product 2-tetradecanol is structurally very similar to the solvent 2-propanol, the two molecules likely interact with the ruthenium catalyst similarly, which would make product inhibition difficult to observe in this system.

Overall, the kinetic data for the **RuPNP** system are consistent with a first-order dependence on [epoxide] and a first-order dependence on [Ru], assumed to be nearly constant in each individual reaction. As described in the Supporting Information, this leads to the second-order rate law shown in eq 1, with *k* equal to 0.167 ± 0.022 M^–1^·s^–1^. This rate constant translates to a standard-state activation free energy of 22.23 ± 0.09 kcal/mol at the reaction temperature of 83 °C, which can be compared to the barrier of 27.1 kcal/mol calculated by DFT.

### RuPNN Kinetics

**Figure 6:**
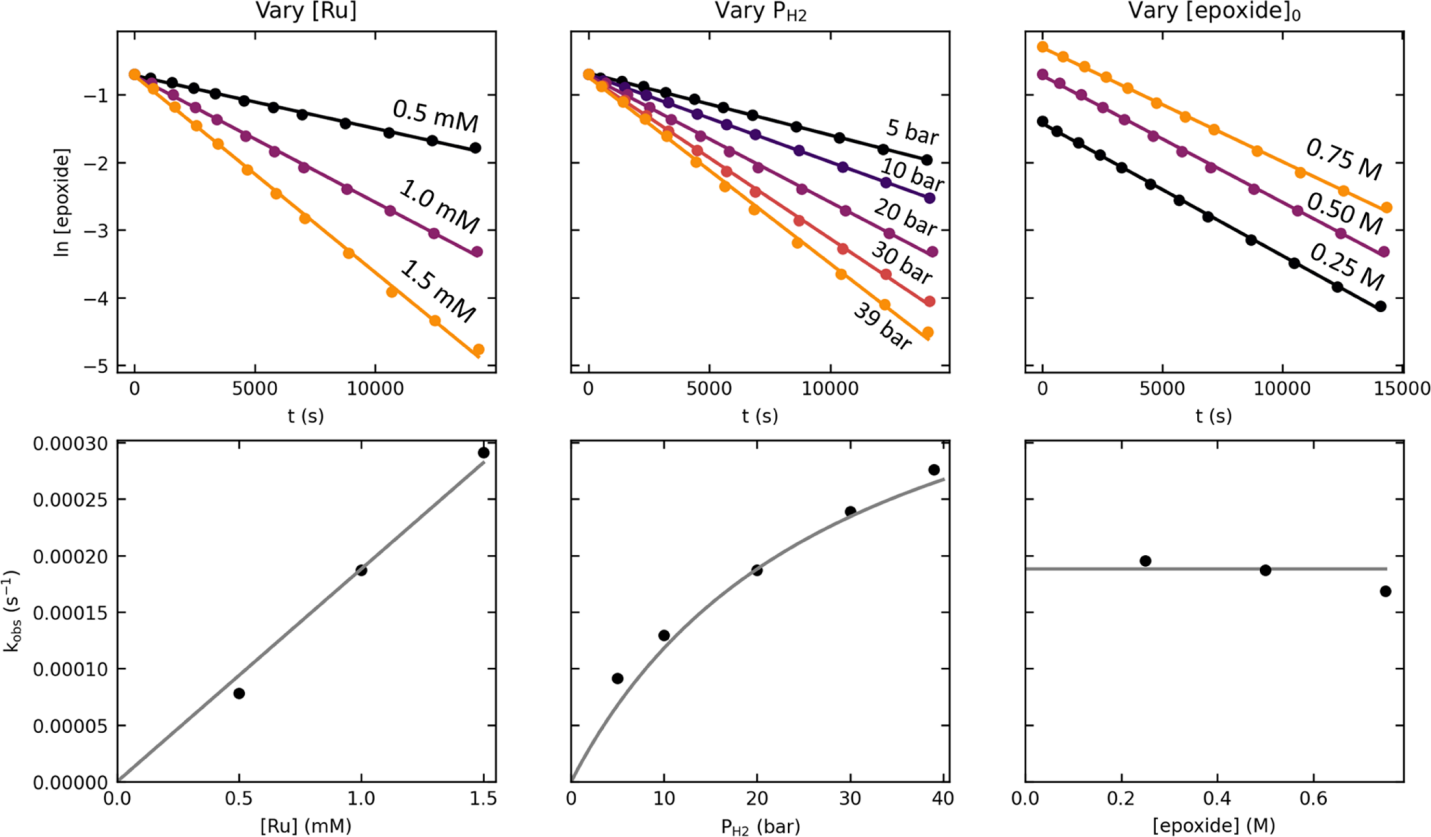
Kinetic data for tetradecene oxide hydrogenolysis catalyzed by **RuPNN**^**imine**^. The top plots (points) show the time course of epoxide conversion using different initial concentrations of the Ru complex, hydrogen pressures, and initial concentrations of the epoxide. The solid lines represent linear fits to the logarithm of [epoxide] over time. The bottom plots show the *k*_obs_ values determined from the slopes of the linear fits (black points). The solid gray lines show the predicted *k*_obs_ values from a global fit of all nine experiments, according to the rate law shown in eq 2.

Figure 6 summarizes the kinetic data for the **RuPNN** system. We again observed a first-order dependence of the rate on [epoxide], as indicated by the linearity of plots of ln[epoxide] vs time. Again, the reaction showed a first-order dependence on [Ru] (left plots), and experiments with different initial [epoxide] showed that saturation in epoxide and product inhibition were minimal (right plots). The dependence of the rate on the hydrogen pressure was not consistent with simple zero- or first-order kinetics but was well-modeled by eq 2, derived from the pre-equilibrium kinetic model shown in Scheme 2. In this model, the steady-state catalyst speciation favors **RuO***^**i**^***Pr**^**PNP**^ at lower hydrogen pressures and **RuH**^**PNP**^ at higher hydrogen pressures, resulting in a nonlinear dependence of the rate on the hydrogen pressure. A global fit of the data gives *K*_1_ = 96.5 ± 35.1 and *k*_2_ = 0.461 ± 0.093 M^–1^·s^–1^. At the reaction temperature of 83 °C, the *K*_1_ value corresponds to a Δ*G*° of −3.23 ± 0.26 kcal/mol for the formation of **RuH**^**PNN**^ and 2-propanol from **RuO***^**i**^***Pr**^**PNN**^ and hydrogen, which can be compared with the value of −6.6 kcal/mol predicted by DFT. The *k*_2_ value corresponds to an activation free energy Δ*G*^‡^ of 21.52 ± 0.14 kcal/mol separating **RuH**^**PNN**^ and **h-TS**^**PNN**^, which can be compared to the value of 25.1 kcal/mol predicted by DFT.

## Discussion

As shown above, the rate laws for both the **RuPNP** and **RuPNN** systems are consistent with the mechanisms calculated by DFT. In both cases, the TDTS is the S_N_2-like epoxide ring-opening transition state **h-TS**. For the **RuPNP** system, the sole TDI is the dihydride complex **RuH**^**PNP**^. For the **RuPNN** system, saturation in hydrogen pressure is observed, which is consistent with a fast pre-equilibrium between the hydridoalkoxide **RuO***^**i**^***Pr**^**PNN**^ and the dihydride **RuH**^**PNN**^, followed by rate-determining epoxide ring-opening through **h-TS**^**PNN**^. In this model, increased hydrogen pressure drives the pre-equilibrium from **RuO***^**i**^***Pr**^**PNN**^ to **RuH**^**PNN**^, causing an increase in the reaction rate.

### Relevance to Prior Mechanistic Proposals

In all of the prior reports of Markovnikov-selective epoxide hydrogenolysis, epoxide ring-opening was proposed to occur through a pathway analogous to Noyori-type bifunctional addition of hydrogen to carbonyl substrates.^17−19^ In the work by Jiao, de Vries, Pignataro, and co-workers,^19^ which employed the Knölker-type iron–cyclopentadienone complex shown in Figure 1, this Noyori-type mechanism was supported through DFT, and an additional pathway through initial Meinwald rearrangement of the epoxide to the aldehyde was found to have a similar barrier. Because our study finds the Noyori-type pathway to have a prohibitively high barrier (see Figure 4, right), it is valuable to critically compare the two studies. First, we note the addition of Lewis acid cocatalysts was an essential element in the work of Jiao, de Vries, Pignataro, and co-workers and was required to obtain the Markovnikov product selectively. Since our work does not employ a cocatalyst, we focus this comparison on catalysis without Lewis acid additives. In this context, two observations are relevant in the prior work: (1) without a Lewis acid cocatalyst, the Knölker-type catalyst gives the anti-Markovnikov (linear) product for hydrogenolysis of aryl epoxides with essentially perfect selectivity for all but two electron-deficient substrates and (2) without a Lewis acid cocatalyst, the Knölker-type catalyst is unreactive toward aliphatic epoxides. So in the absence of a Lewis acid, one would expect a thermally accessible pathway only for the anti-Markovnikov product and only for aryl epoxides. A kinetic study on the hydrogenolysis of styrene oxide gave a pseudo-first-order rate constant of 0.029 min^–1^ at 150 °C,^19^ from which the experimental activation free energy can be estimated as 27.6 kcal/mol. By DFT, the energetic span for the formation of the observed anti-Markovnikov product for styrene oxide is 34.8 kcal/mol for the Meinwald rearrangement pathway and 35.9 kcal/mol for the Noyori-type pathway. In contrast, the energetic span for the formation of the Markovnikov product from styrene oxide is 45.5 kcal/mol. For the model aliphatic substrate 1-butene oxide, energetic spans of 43.3 kcal/mol (Markovnikov) and 42.7 kcal/mol (anti-Markovnikov) can be calculated. Overall, the trends in energy barriers are consistent with the observation that only aryl epoxides are reactive and only the anti-Markovnikov product forms. However, the calculated barriers are significantly higher than the experimental one, and it does not appear that an S_N_2-like pathway analogous to ours was considered in this prior work. Although our current study involves ruthenium instead of iron and does not address the effect of exogenous Lewis acids, we suggest that an S_N_2-like pathway is a plausible alternative in this system as well.

### Is the N–H Group Necessary?

As we described in Introduction, all of the known homogeneous catalysts for Markovnikov-selective epoxide hydrogenolysis have the potential for bifunctional catalysis through the cooperation of a Lewis-acidic metal center and a nearby N–H or O–H group, which may serve as a Brønsted acid or a hydrogen-bond donor. This pattern guided our own search for active catalysts for epoxide hydrogenolysis, but it is worth reevaluating it in light of this mechanistic study. Our DFT calculations indicate that, at least for the **RuPNP** and **RuPNN** systems we studied, the ligand N–H group is not involved in the rate-determining epoxide ring-opening step proceeding through **h-TS**. The N–H group is involved, however, in the pathway for H_2_ activation, through the proton-shuttle transition state **e-TS**. In a previous study on the mechanism of ester hydrogenation catalyzed by **RuH**^**PNN**^, we found that hydrogen cleavage can also be mediated by a CH_2_ linker on the pincer ligand, albeit with a higher barrier.^9l^ In a computational study of Milstein’s catalyst, which is structurally similar to **RuH**^**PNN**^ but features an NEt_2_ group instead of an NHEt group, Gusev showed that heterolytic hydrogen cleavage can be mediated by an exogenous alkoxide base, without the involvement of the pincer ligand.^23b^ In light of these observations, we suggest that it may be possible to identify catalysts for Markovnikov-selective epoxide hydrogenolysis that lack the capacity for Noyori/Shvo-type bifunctional catalysis, which may allow for the development of systems with different or improved selectivity. In particular, no homogeneous catalyst has been reported that promotes the Markovnikov-selective hydrogenolysis of enantiomerically enriched epoxides without racemization of the product secondary alcohol. Because product racemization likely proceeds through reversible dehydrogenation of the alcohol to the ketone, it may be possible to alleviate this side reaction through judicious catalyst design.

## Conclusions

In summary, we report a combined computational/experimental study on the mechanism of Markovnikov-selective epoxide hydrogenolysis, catalyzed by RuPNP and RuPNP complexes. We find that in both systems, heterolytic hydrogen cleavage proceeds through the expected ligand-assisted pathway and requires a molecule of 2-propanol to act as a proton shuttle. In contrast, epoxide ring-opening proceeds through an S_N_2-like nucleophilic attack of the ruthenium hydride on the terminal carbon of the epoxide, without the involvement of the ligand N–H groups. We are actively applying these mechanistic findings toward the development of catalyst systems with improved reactivity and selectivity.

## Supporting Information Available

Experimental and computational details (PDF)
Atomic coordinates (*XYZ*) for all computed molecular structures, compiled as one file readable by the free program Mercury^28^ (XYZ)

## Supplementary Material

om2c00503_si_001.pdf

om2c00503_si_002.xyz
