# Supplementary material for: The Mechanism of Markovnikov-Selective Epoxide Hydrogenolysis Catalyzed by Ruthenium PNN and PNP Pincer Complexes
Source: Organometallics. 2023 Feb 27;42(5):347–56. doi: 10.1021/acs.organomet.2c00503 (PMC10015984; doi:10.1021/acs.organomet.2c00503)
Supplement: Supplementary file 1 — om2c00503_si_001.pdf [file om2c00503_si_001.pdf]

Supporting Information for

The Mechanism of Markovnikov-Selective Epoxide Hydrogenolysis Catalyzed by Ruthenium PNN and PNP Pincer Complexes

Marianna C. Head, Benjamin T. Joseph, Jason M. Keith*, and Anthony R. Chianese*

Department of Chemistry, Colgate University, 13 Oak Drive, Hamilton, New York 13346, United States

*Email addresses: J.M. Keith: jkeith@colgate.edu; A.R. Chianese: achianese@colgate.edu

Table of Contents

General Methods. 1

Computational Methods. 1

Consideration of potential TDIs, including explicit solvation. 2

Alternative pathways: Hydrogen activation without a proton shuttle. 4

Alternative pathways: Epoxide ring-opening including an explicit solvent molecule. 4

Energies Calculated by DFT. 5

Apparatus for kinetic experiments. 10

Experimental procedure for kinetic experiments. 10

Table S3. Concentration data from kinetic experiments, RuPNP catalyst. 11

Table S4. Concentration data from kinetic experiments, RuPNN catalyst. 13

Determination of the rate law and activation barrier for the RuPNP catalyst. 15

Determination of the rate law and activation barrier for the RuPNN catalyst. 16

References 20

General Methods. All epoxide hydrogenolysis reactions were assembled in an argon-filled MBraun Labmaster 130 glovebox. Solvents were purchased in anhydrous form from EMD-Millipore or Acros and were deoxygenated by sparging with argon before bringing into the glovebox. All reagents and materials were commercially available and were used as received, unless otherwise noted. Hydrogen gas was purchased from Airgas at the Ultrahigh Purity level. Gas chromatography was conducted using a Shimadzu 2030 system equipped with an FID detector. **RuPNN^{imine}** was synthesized as previously described.[1] **Ru-MACHO-BH** was purchased from Strem Chemicals. Tetradecene oxide and tetradecane were purchased from TCI Chemicals and were sparged with argon in oven-dried flasks for 45 minutes before bringing into the glovebox.

Computational Methods. Density functional theory calculations were performed using the Gaussian 16 computational chemistry package, Revision B.01.[2] The geometries and energies of all species were calculated using the dispersion-corrected hybrid functional ωB97X-D,[3] and the def2-SVP basis set.[4] A superfine integration

grid was used for all calculations, which aided convergence of structures with loosely bound fragments such as explicit isopropyl alcohol molecules. Complete structures with no truncations were used in all cases. Geometry optimization and frequency calculation were conducted in solvent, using a polarizable continuum with radii and non-electrostatic terms from Truhlar and coworkers' SMD solvation model, and with dielectric constants chosen for 2-propanol.[5] Geometry optimization in solvent is important to identify ion-pair intermediates that might be missed in the gas phase.[6] However, convergence is occasionally problematic. In difficult cases, we repeated the calculations using different, nearly converged starting points, each time with a fresh calculation of the force constants, using a range of values for the maximum step size. Despite this effort, convergence in solvent was not successful for **RuO^{i}Pr-solv^{PNN}** and **a-TS^{PNN}**. For these structures, the geometry optimization and frequency calculation used for free-energy corrections were done in the gas phase at the same ωB97X-D/def2-SVP level described above, and a single-point electronic-energy refinement at the ωB97X-D/def2-TZVP level was conducted using the solvent model.

Frequency calculations ensured the absence of imaginary vibrational modes in intermediates and the presence of exactly one imaginary mode in transition states. Intrinsic reaction coordinate calculations were employed to verify that transition states led to the specified minima. Free-energy corrections were calculated at the experimental reaction temperature of 83 °C, or 356.15 K. Standard state corrections were added in order to adjust from 1 atm to 1 M for solution-phase free energies, amounting to 2.39 kcal/mol added to the free energy of each isolated molecule at 356.15 K.[7] Although the standard state for molecular hydrogen is sometimes taken as the gas at 1 atm, we have used a 1 M standard state in 2-propanol, for consistency in computing reaction kinetics from the calculated free energies. The solvation-corrected electronic energies were further refined using the same ωB97X-D functional and the larger basis set def2-TZVP.[4] All free energies reported in the paper refer to 1.0 M standard concentrations at 356.15 K. A table of energies is provided in the Supporting Information, and geometries in Cartesian coordinates are included in a separate, compiled .XYZ file. The .XYZ file also contains the single imaginary frequency for all transition states identified.

To assess the dependence of the calculated energies on the selected density functional, we have recalculated electronic single-point energies using the same def2-TZVP basis set and three additional functionals: B3LYP-D3[8] (applying Grimme's D3 dispersion correction),[9] M06L,[10] and MN15.[11] Table S2 shows the effect of functional choice on the relative free energies. Broadly, the effect of functional choice is minimal and does not change the key conclusions regarding the overall catalytic barriers or the identities of turnover-frequency-determining species.

Consideration of potential TDIs, including explicit solvation. In the early stages of the development of the MEPs shown in Figure 2 in the main text, we compared the free energies of plausible catalyst resting states. Figure S1 below shows the relative free energies of alkoxide **RuO^{i}Pr**, unsaturated species **c-nosolv**, and hydride **RuH** for the **RuPNP** and **RuPNN** systems. In a previous study on the mechanism of ester hydrogenation catalyzed by **RuH^{PNN}**, we found it essential to consider an additional hydrogen-bonded alcohol molecule to correctly model catalyst speciation.[1b] For each intermediate in this work, we considered the effect of including a molecule of 2-propanol, the optimal solvent for the reaction and the hydrogenation product for our model substrate, propylene oxide. We note that for the **RuPNN** system, convergence of **RuO^{i}Pr-solv** in continuum solvent was unsuccessful despite multiple attempts. This species was optimized in the gas phase, and the gas-phase free-energy correction was applied to the single-point electronic energy calculated using continuum solvent. Each species (**RuO^{i}Pr-solv**, **c**, and **f**) shows a small (≤3.1 kcal/mol) effect of adding an explicit hydrogen-bonded 2-propanol molecule. This contrasts with our previous study, which showed that an explicit ethanol molecule stabilized the ethoxide intermediate

analogous to **RuO^{i}Pr** by 7.4 kcal/mol, resulting in an alkoxide resting state for that system. This difference can be rationalized by considering the solvents used: the prior study employed toluene in both the computational and kinetic studies, while the present study employs 2-propanol as solvent. In the more polar 2-propanol solvent model, the inclusion of explicit hydrogen bonding has a smaller effect on the calculated energies. Because none of the 2-propanol adducts **RuO^{i}Pr-solv**, **c**, and **f** were significantly more stable than their unsolvated counterparts **RuO^{i}Pr**, **c-nosolv**, and **RuH**, the 2-propanol adducts were not considered in the kinetic modeling described later. Note that, as shown in Figure 2, **c** rather than **c-nosolv** lies on the MEP because hydrogen activation is substantially facilitated by an alcohol proton shuttle.

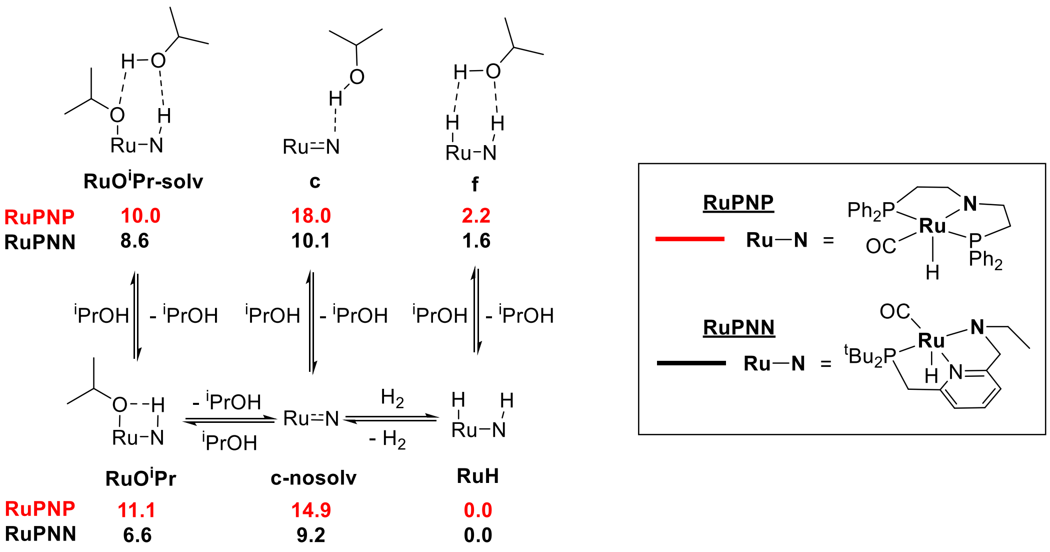

Figure S1. Comparison of plausible resting states for the **RuPNP** and **RuPNN** catalysts. Energies given are free energies at the experimental reaction temperature of 356.15 K, corrected to a 1.0 M concentration.

For both the **RuPNP** and **RuPNN** systems, we find that the hydride species **RuH** is the most stable at the 1 M standard state. The unsaturated species **c-nosolv** is high enough in energy that it does not represent a significant fraction of the resting speciation for either catalyst. In the **RuPNP** system, the alkoxide species **RuO^{i}Pr** also represents a minor component in catalyst speciation, so the hydride **RuH** can be considered to be the dominant resting state and the sole turnover frequency-determining intermediate (TDI). The situation is potentially more complicated for the **RuPNN** system because of the lower free energy of the alkoxide intermediate **RuO^{i}Pr^{PNN}**. Assuming a fast equilibrium under catalytic conditions, the calculated free energy difference of 6.6 kcal/mol between **RuO^{i}Pr^{PNN}** and **e** corresponds to an **RuO^{i}Pr^{PNN}**: **RuH^{PNN}** ratio of 1:99, given a neat molarity of 13.1 M for 2-propanol and a hydrogen solubility of 0.093 M at 20 bar and 83 °C (see Equation S1 below). However, if the free energy difference were overestimated by the DFT method, **RuO^{i}Pr^{PNN}** would represent a greater fraction of the resting catalyst speciation. Figure S2 shows the predicted fraction of **RuO^{i}Pr^{PNN}** as a function of its free energy relative to **RuH^{PNN}** under these conditions. If **RuO^{i}Pr^{PNN}** lies above **RuH^{PNN}** by only 3.5 kcal/mol for example, a 1:1 ratio of the two species is expected. In this regime, the steady-state concentration of **e** is expected to be influenced by the hydrogen concentration, which would result in saturation kinetics, as derived on page S14.

$$\text{(S1)} \qquad \mathbf{RuO^iPr^{PNN}} + H_2 \rightleftharpoons \mathbf{RuH^{PNN}} + HO^iPr \qquad K = \frac{[\mathbf{RuH^{PNN}}][HO^iPr]}{[\mathbf{RuO^iPr^{PNN}}][H_2]}$$

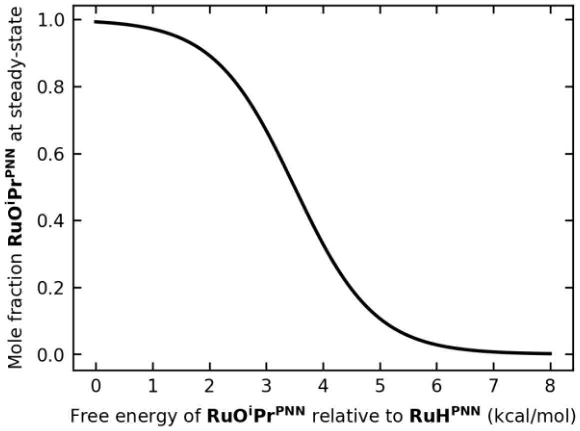

Figure S2. Predicted fraction of **RuO^{i}Pr^{PNN}** as a function of its free energy relative to **RuH^{PNN}**.

Alternative pathways: Hydrogen activation without a proton shuttle. In the MEPs shown in Figure 2, hydrogen activation is assisted by a molecule of 2-propanol, giving a relative TS free energy of 22.6 kcal/mol for the **RuPNP** catalyst and 19.1 for the **RuPNN** catalyst. For comparison, we calculated the analogous pathways without a proton shuttle and find higher barriers. Figure S3 shows these sequences, with the **RuPNP** system in red and the **RuPNN** system in black. Without a proton shuttle, we find significantly higher barriers of 32.2 kcal/mol for the **RuPNP** system and 28.8 kcal/mol for the **RuPNN** system.

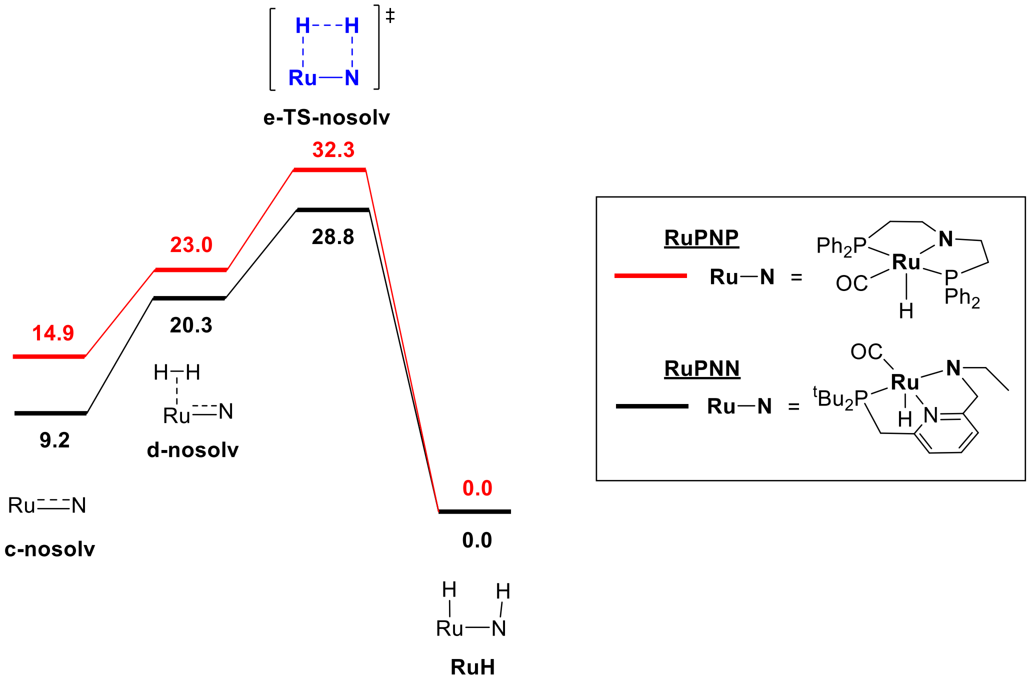

Figure S3. Hydrogen activation without a proton shuttle. The **RuPNP** pathway is shown in red and the **RuPNN** pathway is shown in black. Free energies are in kcal/mol against the hydride resting state **RuH**.

Alternative pathways: Epoxide ring-opening including an explicit solvent molecule. In the MEPs shown in Figure 2, epoxide ring-opening is modeled without an explicit solvent molecule. Because the epoxide oxygen is developing a negative charge in **h-TS**, we considered that an explicit 2-propanol might be necessary to properly

model this species. Figure S4 shows the epoxide ring-opening sequence for the **RuPNN** system with an isopropanol molecule included to stabilize the developing negative charge on the oxygen atom. The relative free energy of **h-TS-solv^{PNN}** is 31.0, which can be compared with the free energy of 25.1 for **h-TS^{PNN}**, the analogous TS lacking an explicit solvent molecule.

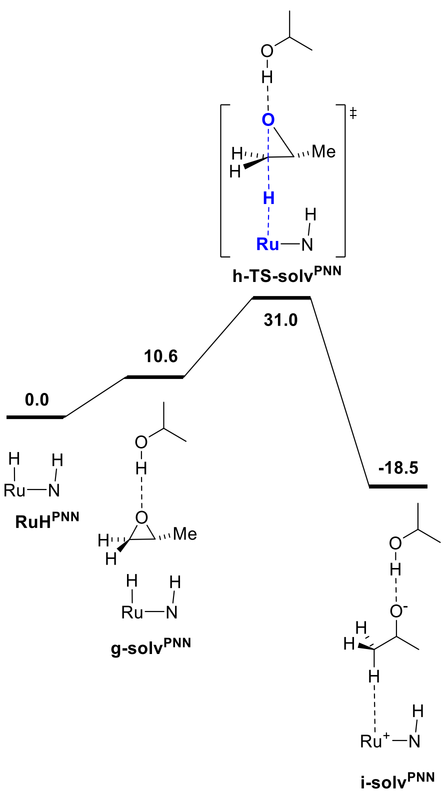

Figure S4. Epoxide ring-opening pathway for **RuPNN** system, including an explicit 2-propanol molecule to stabilize the developing negative charge on the epoxide oxygen.

Energies Calculated by DFT. Table S1 below shows the energies calculated by DFT for all structures reported in this paper. The column **E(BS2)** represents the solvent-corrected electronic energy in hartrees, calculated with the ωB97X-D functional and the def2-TZVP basis set. The column **G(corr)** represents the correction to the Gibbs free energy calculated at 356.15 K after geometry optimization using the ωB97X-D functional and the def2-SVP basis set. The column **SS G (kcal)** represents the Gibbs free energy for each isolated species at 356.15 K in kcal/mol, including the addition of 2.39 kcal/mol for each molecule to convert to a 1 M concentration. The column **Mass Balance** lists the small molecules included in the total free energy for the calculation of reaction pathways. The column **G(total, kcal)** is the sum of free energies of the ruthenium complex and any small molecules included for mass balance. The column **G(rel)** is the total free energy referenced against **e** with the organic reactants.

Table S2 shows the solvent-corrected single-point electronic energies calculated by DFT using ωB97X-D in addition to three other density functionals for comparison: B3LYP-D3, M06L, and MN15 (left), along with the relative free energies for each functional. All free energies employ the same free-energy correction calculated at the ωB97X-D/def2-SVP level as described above.

Table S1. Energies calculated by DFT.

Small Molecules	E(BS2)	G(corr)	SS G (kcal)
hydrogen	-1.175828682	-0.004453	-738.25
propylene_oxide	-193.1307711	0.05333	-121155.44
2-propanol	-194.387128	0.074392	-121930.60
2-propoxide	-193.8648524	0.059937	-121611.94
1-propanol	-194.3815428	0.074506	-121927.03

RuPNP Resting State Speciation				Mass Balance	G(total, kcal)	G(rel)
RuOiPr^{PNP}	-2224.784189	0.527496	-1395738.71	hydrogen, propylene_oxide, 2-propanol	-1639563.00	11.07
RuOiPr-solv^{PNP}	-2419.194555	0.627173	-1517670.41	hydrogen, propylene_oxide	-1639564.11	9.97
c-nosolv^{PNP}	-2030.363489	0.421822	-1273804.28	hydrogen, propylene_oxide, 2 * 2-propanol	-1639559.18	14.90
c^{PNP}	-2224.768439	0.522731	-1395731.81	hydrogen, propylene_oxide, 2-propanol	-1639556.11	17.96
RuH^{PNP}	-2031.584653	0.442771	-1274557.42	propylene_oxide, 2 * 2-propanol	-1639574.08	0.00
f^{PNP}	-2225.987245	0.539891	-1396485.86	propylene_oxide, 2-propanol	-1639571.90	2.17

RuPNP – MEP						
RuOiPr^{PNP}	-2224.784189	0.527496	-1395738.71	hydrogen, propylene_oxide, 2-propanol	-1639563.00	11.07
a-TS^{PNP}	-2224.766301	0.519768	-1395732.33	hydrogen, propylene_oxide, 2-propanol	-1639556.63	17.45
b^{PNP}	-2224.769218	0.523663	-1395731.72	hydrogen, propylene_oxide, 2-propanol	-1639556.02	18.06
c^{PNP}	-2224.768439	0.522731	-1395731.81	hydrogen, propylene_oxide, 2-propanol	-1639556.11	17.96
d^{PNP}	-2225.953727	0.538519	-1396465.68	propylene_oxide, 2-propanol	-1639551.73	22.34
e-TS^{PNP}	-2225.949993	0.535251	-1396465.39	propylene_oxide, 2-propanol	-1639551.44	22.64
f^{PNP}	-2225.987245	0.539891	-1396485.86	propylene_oxide, 2-propanol	-1639571.90	2.17
RuH^{PNP}	-2031.584653	0.442771	-1274557.42	propylene_oxide, 2 * 2-propanol	-1639574.08	0.00
g^{PNP}	-2224.726831	0.52084	-1395706.89	2 * 2-propanol	-1639568.10	5.98
h-TS^{PNP}	-2224.693616	0.521218	-1395685.81	2 * 2-propanol	-1639547.02	27.06
i^{PNP}	-2224.749446	0.525613	-1395718.09	2 * 2-propanol	-1639579.29	-5.22
j^{PNP}	-2030.863989	0.438292	-1274108.01	2 * 2-propanol + 2-propoxide	-1639581.16	-7.08
RuOiPr^{PNP}	-2224.784189	0.527496	-1395738.71	2 * 2-propanol	-1639599.91	-25.84

RuPNP - H_2 activation without proton shuttle						
c-nosolv^{PNP}	-2030.363489	0.421822	-1273804.28	hydrogen, propylene_oxide, 2 * 2-propanol	-1639559.18	14.90
d-nosolv^{PNP}	-2031.544329	0.439041	-1274534.46	propylene_oxide, 2 * 2-propanol	-1639551.11	22.96
e-TS-nosolv^{PNP}	-2031.526445	0.436011	-1274525.14	propylene_oxide, 2 * 2-propanol	-1639541.79	32.28
RuH^{PNP}	-2031.584653	0.442771	-1274557.42	propylene_oxide, 2 * 2-propanol	-1639574.08	0.00

RuPNP - Epoxide ring-opening toward linear alcohol						
g-linear^{PNP}	-2224.726957	0.519625	-1395707.73	2 * 2-propanol	-1639568.94	5.14
h-TS-linear^{PNP}	-2224.687619	0.521809	-1395681.68	2 * 2-propanol	-1639542.88	31.19
i-linear^{PNP}	-2224.744946	0.525592	-1395715.28	2 * 2-propanol	-1639576.48	-2.41

RuPNP - Noyori-type concerted hydride/proton transfer						
g-Noyori^{PNP}	-2224.729723	0.519383	-1395709.62	2 * 2-propanol	-1639570.83	3.25
h-TS-Noyori^{PNP}	-2224.655199	0.519718	-1395662.64	2 * 2-propanol	-1639523.85	50.22
i-Noyori^{PNP}	-2224.770909	0.524251	-1395732.41	2 * 2-propanol	-1639593.62	-19.54

RuPNN Resting State Speciation

RuOiPr^{PNN}	-1520.217269	0.499837	-953633.98	hydrogen, propylene_oxide, 2-propanol	-1197458.28	6.55
RuOiPr-solv^{PNN}	-1714.623822	0.600799	-1075562.49	hydrogen, propylene_oxide	-1197456.18	8.65
c-nosolv^{PNN}	-1325.797486	0.393139	-831700.77	hydrogen, propylene_oxide, 2 * 2-propanol	-1197455.67	9.16
c^{PNN}	-1520.203856	0.49201	-953630.47	hydrogen, propylene_oxide, 2-propanol	-1197454.77	10.06
RuH^{PNN}	-1327.012681	0.417265	-832448.18	propylene_oxide, 2 * 2-propanol	-1197464.83	0.00
f^{PNN}	-1521.413973	0.512162	-954377.19	propylene_oxide, 2-propanol	-1197463.24	1.59

RuPNN - MEP

RuOiPr^{PNN}	-1520.217269	0.499837	-953633.98	hydrogen, propylene_oxide, 2-propanol	-1197458.28	6.55
a-TS^{PNN}	-1520.196464	0.494	-953624.59	hydrogen, propylene_oxide, 2-propanol	-1197448.89	15.94
b^{PNN}	-1520.200871	0.495206	-953626.59	hydrogen, propylene_oxide, 2-propanol	-1197450.89	13.93
c^{PNN}	-1520.203856	0.49201	-953630.47	hydrogen, propylene_oxide, 2-propanol	-1197454.77	10.06
d^{PNN}	-1521.384287	0.510698	-954359.48	propylene_oxide, 2-propanol	-1197445.53	19.30
e-TS^{PNN}	-1521.382208	0.508248	-954359.71	propylene_oxide, 2-propanol	-1197445.76	19.07
f^{PNN}	-1521.413973	0.512162	-954377.19	propylene_oxide, 2-propanol	-1197463.24	1.59
RuH^{PNN}	-1327.012681	0.417265	-832448.18	propylene_oxide, 2 * 2-propanol	-1197464.83	0.00
g^{PNN}	-1520.152794	0.490962	-953599.09	2 * 2-propanol	-1197460.30	4.53
h-TS^{PNN}	-1520.120621	0.491532	-953578.54	2 * 2-propanol	-1197439.75	25.08
i^{PNN}	-1520.186947	0.496961	-953616.76	2 * 2-propanol	-1197477.96	-13.14
j^{PNN}	-1326.305013	0.410659	-832008.25	2 * 2-propanol + 2-propoxide	-1197481.40	-16.57
RuOiPr^{PNN}	-1520.217269	0.499837	-953633.98	2 * 2-propanol	-1197495.19	-30.36

RuPNN - H_2 activation without proton shuttle

c-nosolv^{PNN}	-1325.797486	0.393139	-831700.77	hydrogen, propylene_oxide, 2 * 2-propanol	-1197455.67	9.16
d-nosolv^{PNN}	-1326.974358	0.411274	-832427.89	propylene_oxide, 2 * 2-propanol	-1197444.54	20.29
e-TS-nosolv^{PNN}	-1326.958637	0.409109	-832419.38	propylene_oxide, 2 * 2-propanol	-1197436.03	28.80
RuH^{PNN}	-1327.012681	0.417265	-832448.18	propylene_oxide, 2 * 2-propanol	-1197464.83	0.00

RuPNN - SN2 epoxide ring opening with explicit solvent

RuH^{PNN}	-1327.012681	0.417265	-832448.18	propylene_oxide, 2 * 2-propanol	-1197464.83	0.00
g-solv^{PNN}	-1714.553777	0.592673	-1075523.63	2-propanol	-1197454.24	10.59
h-TS-solv^{PNN}	-1714.522966	0.594393	-1075503.22	2-propanol	-1197433.82	31.01
i-solv^{PNN}	-1714.600929	0.593416	-1075552.75	2-propanol	-1197483.36	-18.53

RuPNN - Epoxide ring-opening toward linear alcohol

g-linear^{PNN}	-1520.153505	0.492569	-953598.53	2 * 2-propanol	-1197459.73	5.09
h-TS-linear^{PNN}	-1520.115124	0.493135	-953574.09	2 * 2-propanol	-1197435.30	29.53
i-linear^{PNN}	-1520.182491	0.493845	-953615.92	2 * 2-propanol	-1197477.12	-12.29

RuPNP - Noyori-type concerted hydride/proton transfer

g-Noyori^{PNN}	-1520.153997	0.488405	-953601.45	2 * 2-propanol	-1197462.66	2.17
h-TS-Noyori^{PNN}	-1520.082642	0.490186	-953555.56	2 * 2-propanol	-1197416.76	48.07
b^{PNN}	-1520.200871	0.495206	-953626.59	2 * 2-propanol	-1197487.80	-22.97

Table S2. Energies calculated by DFT using the functionals ωB97XD, B3LYP-D3, M06L, and MN15.

Small Molecules	E(B3LYP-D3)	E(M06L)	E(MN15)
hydrogen	-1.179308116	-1.171323036	-1.168609623
propylene_oxide	-193.200128	-193.1659412	-192.9629721
2-propanol	-194.4585414	-194.4100705	-194.206516
2-propoxide	-193.939695	-193.8903868	-193.6884452
1-propanol	-194.4527069	-194.4045739	-194.2002605

				G(rel, kcal/mol)			
**RuPNP Resting State Speciation**				**ωB97XD**	**B3LYP-D3**	**M06L**	**MN15**
RuOiPr^{PNP}	-2225.4002	-2225.068283	-2223.069041	11.07	7.75	8.82	12.34
RuOiPr-solv^{PNP}	-2419.881912	-2419.496669	-2417.296815	9.97	6.69	10.81	12.48
c-nosolv^{PNP}	-2030.913224	-2030.627681	-2028.831138	14.90	8.35	10.74	14.79
c^{PNP}	-2225.390658	-2225.053034	-2223.051589	17.96	10.74	15.40	20.30
RuH^{PNP}	-2032.127432	-2031.837714	-2030.044919	0.00	0.00	0.00	0.00
f^{PNP}	-2226.602563	-2226.261248	-2224.26417	2.17	1.46	3.43	3.88
**RuPNP - Epoxide hydrogenation MEP**							
RuOiPr^{PNP}	-2225.4002	-2225.068283	-2223.069041	11.07	7.75	8.82	12.34
a-TS^{PNP}	-2225.383345	-2225.05045	-2223.053171	17.45	13.47	15.16	17.45
b^{PNP}	-2225.386084	-2225.054807	-2223.056803	18.06	14.20	14.87	17.61
c^{PNP}	-2225.390658	-2225.053034	-2223.051589	17.96	10.74	15.40	20.30
d^{PNP}	-2226.572537	-2226.23197	-2224.232986	22.34	19.45	20.94	22.59
e-TS^{PNP}	-2226.569215	-2226.225718	-2224.227126	22.64	19.48	22.81	24.22
f^{PNP}	-2226.602563	-2226.261248	-2224.26417	2.17	1.46	3.43	3.88
RuH^{PNP}	-2032.127432	-2031.837714	-2030.044919	0.00	0.00	0.00	0.00
g^{PNP}	-2225.337943	-2225.011134	-2223.019958	5.98	6.62	8.44	5.56
h-TS^{PNP}	-2225.314836	-2224.98041	-2222.982277	**27.06**	**21.36**	**27.96**	**29.45**
i^{PNP}	-2225.369686	-2225.031456	-2223.031361	-5.22	-10.30	-1.31	1.40
j^{PNP}	-2031.411336	-2031.121429	-2029.319255	-7.08	-13.39	-3.79	1.46
RuOiPr^{PNP}	-2225.4002	-2225.068283	-2223.069041	-25.84	-28.27	-23.24	-21.06
**RuPNP - H_2 activation without proton shuttle**							
c-nosolv^{PNP}	-2030.913224	-2030.627681	-2028.831138	14.90	8.35	10.74	14.79
d-nosolv^{PNP}	-2032.091104	-2031.804223	-2030.007793	22.96	20.45	18.68	20.96
e-TS-nosolv^{PNP}	-2032.072751	-2031.785645	-2029.988581	32.28	30.07	28.43	31.11
RuH^{PNP}	-2032.127432	-2031.837714	-2030.044919	0.00	0.00	0.00	0.00
**RuPNP - Epoxide ring-opening toward linear alcohol**							
g-linear^{PNP}	-2225.338349	-2225.012272	-2223.020559	5.14	5.60	6.97	4.43
h-TS-linear^{PNP}	-2225.30936	-2224.975083	-2222.976409	31.19	25.17	31.67	33.50
i-linear^{PNP}	-2225.365178	-2225.02769	-2223.026005	-2.41	-7.49	1.04	4.75
**RuPNP - Noyori-type concerted hydride/proton transfer**							
g-Noyori^{PNP}	-2225.341636	-2225.015078	-2223.020798	3.25	3.39	5.05	4.12
h-TS-Noyori^{PNP}	-2225.279718	-2224.943794	-2222.94476	**50.22**	**42.45**	**50.00**	**52.05**
i-Noyori^{PNP}	-2225.393125	-2225.053889	-2223.053217	-19.54	-25.87	-16.24	-13.17

					G(rel, kcal/mol)		
**RuPNN Resting State Speciation**				**ωB97XD**	**B3LYP-D3**	**M06L**	**MN15**
RuOiPr^{PNN}	-1520.65336	-1520.415724	-1518.903158	6.55	4.13	4.56	7.43
RuOiPr-solv^{PNN}	-1715.130593	-1714.841513	-1713.126163	8.65	6.69	8.98	11.37
c-nosolv^{PNN}	-1326.16806	-1325.975436	-1324.662723	9.16	3.04	5.63	10.83
c^{PNN}	-1520.645796	-1520.402223	-1518.887307	10.06	3.97	8.12	12.47
RuH^{PNN}	-1327.376987	-1327.180511	-1325.873369	0.00	0.00	0.00	0.00
f^{PNN}	-1521.850307	-1521.602094	-1520.090849	1.59	1.21	3.26	3.60

**RuPNN - Epoxide hydrogenation MEP**							
RuOiPr^{PNN}	-1520.65336	-1520.415724	-1518.903158	6.55	4.13	4.56	7.43
a-TS^{PNN}	-1520.632421	-1520.394075	-1518.883222	15.94	13.61	14.48	16.28
b^{PNN}	-1520.638467	-1520.400025	-1518.886088	13.93	10.57	11.50	15.24
c^{PNN}	-1520.645796	-1520.402223	-1518.887307	10.06	3.97	8.12	12.47
d^{PNN}	-1521.823365	-1521.575928	-1520.062149	19.30	17.19	18.76	20.69
e-TS^{PNN}	-1521.821357	-1521.571669	-1520.058367	19.07	16.92	19.89	21.53
f^{PNN}	-1521.850307	-1521.602094	-1520.090849	1.59	1.21	3.26	3.60
RuH^{PNN}	-1327.376987	-1327.180511	-1325.873369	0.00	0.00	0.00	0.00
g^{PNN}	-1520.585543	-1520.352652	-1518.844889	4.53	5.11	6.50	5.03
h-TS^{PNN}	-1520.562581	-1520.321942	-1518.808943	**25.08**	**19.87**	**26.13**	**27.94**
i^{PNN}	-1520.626896	-1520.381319	-1518.866256	-13.14	-17.08	-7.72	-4.61
j^{PNN}	-1326.671967	-1326.475971	-1325.160419	-16.57	-21.68	-12.49	-7.86
RuOiPr^{PNN}	-1520.65336	-1520.415724	-1518.903158	-30.36	-31.88	-27.51	-25.97

**RuPNN - H_2 activation without proton shuttle**							
c-nosolv^{PNN}	-1326.16806	-1325.975436	-1324.662723	9.16	3.04	5.63	10.83
d-nosolv^{PNN}	-1327.34209	-1327.148173	-1325.836989	20.29	18.14	16.53	19.07
e-TS-nosolv^{PNN}	-1327.326214	-1327.131761	-1325.819914	28.80	26.74	25.47	28.43
RuH^{PNN}	-1327.376987	-1327.180511	-1325.873369	0.00	0.00	0.00	0.00

**RuPNN - SN2 epoxide ring opening with explicit solvent**							
g-solv^{PNN}	-1715.057601	-1714.774764	-1713.065002	10.59	11.38	13.70	11.25
h-TS-solv^{PNN}	-1715.035462	-1714.745573	-1713.030707	31.01	26.35	33.10	33.85
i-solv^{PNN}	-1715.113641	-1714.815842	-1713.098666	-18.53	-23.32	-11.61	-9.40

**RuPNN - Epoxide ring-opening toward linear alcohol**							
g-linear^{PNN}	-1520.586071	-1520.353327	-1518.845554	5.09	5.78	7.09	5.62
h-TS-linear^{PNN}	-1520.557456	-1520.318403	-1518.804117	29.53	24.09	29.36	31.98
i-linear^{PNN}	-1520.623032	-1520.379119	-1518.86137	-12.29	-16.61	-8.30	-3.50

**RuPNP - Noyori-type concerted hydride/proton transfer**							
g-Noyori^{PNN}	-1520.587617	-1520.354864	-1518.845469	2.17	2.20	3.51	3.06
h-TS-Noyori^{PNN}	-1520.528327	-1520.286568	-1518.770733	**48.07**	**40.52**	**47.48**	**51.08**
b^{PNN}	-1520.638467	-1520.400025	-1518.886088	-22.97	-25.44	-20.56	-18.16

Apparatus for kinetic experiments. Kinetic experiments were conducted in an Asynt Multicell Parallel High-Pressure Reactor, designed to allow sampling of aliquots from five hydrogenation reactions run in parallel. Our customization of this apparatus was described previously,[1a] but is repeated here for convenience. The reactor was customized to fit in our glovebox antechamber and to include reaction sampling valves on five reactor cells. The sampling valves employ 0.8 mm ID 1/16” stainless steel tubing, a Swagelok Low Flow metering valve (part no. SS-SS1) to control flow, and a Swagelok ball valve (part no. SS-41GS1) to allow removal of samples. As the internal volume of the sampling valve system was measured to be 0.25 mL, removal of 0.50 mL of liquid before collecting each aliquot ensures that a fresh sample was being taken directly from the reaction mixture. A sixth reactor cell is fitted with a thermocouple to allow control of the internal reaction temperatures. For heated reactions, the reactor was partially submerged (2 cm past the internal liquid level) in a stirred oil bath (a non-magnetic stainless-steel cooking pot), whose temperature is controlled using heating tape (Briskheat part no. BIH051040L) wrapped around the oil bath and an Omega Platinum Series Universal Benchtop Digital Controller, part no. CS8DPT. The oil bath is placed on a stirring hotplate, which allows stirring of the reactions and oil bath, as well as pre-heating of the oil to allow the reactions equilibrate to the desired temperature quickly.

Experimental procedure for kinetic experiments. First, the oil bath was preheated to an empirically determined temperature above the desired reaction temperature to enable rapid equilibration to the desired temperature (90 °C for the 83 °C reactions described in this work). The Asynt reactor was brought into the glovebox with oven-dried glass reactor liners and Teflon-coated stir bars. Reaction solutions, with a total volume of 10.0 mL, were prepared with the appropriate amount of ruthenium complex, epoxide, and tetradecane (0.20 equiv. relative to epoxide) as internal standard. The reactor was closed and removed from the glovebox and allowed to incubate for 20 minutes in the water bath. After gently purging the H_2 line for 3 minutes, the reactor was pressurized to bar the desired pressure. The thermocouple was connected to the Omega temperature controller, and the reactor was submerged in the pre-heated oil bath with stirring set to 500 rpm. The internal reaction temperature was recorded each minute until it was stable, and time zero was marked when the temperature was 5 °C below the target temperature. Typically, the warmup time was 5-10 minutes. Aliquots were removed at predetermined times for analysis by gas chromatography. To ensure that samples represented the reaction mixture without contamination from the transfer line, 0.5 mL of reaction mixture was discarded before one drop was collected for each aliquot. Each one-drop aliquot was diluted with 1 mL toluene in a crimp-top vial, then sealed and analyzed by GC-FID. The concentrations of reactant and products at each time point were determined by integration of their GC signals against the tetradecane standard. GC analysis employed a Shimadzu SH-Rxi-5ms column 15 meters in length. The oven temperature was initially held at 50 °C for 3 minutes, then increased to 250 °C at a rate of 20 °C/min, then held at 250 °C for 2 minutes. With these parameters, tetradecene oxide eluted at 10.09 minutes, 2-tetradecanol eluted at 9.98 minues, 1-tetradecanol (less than 1% of the product) eluted at 10.38 minutes, and the standard tetradecane eluted at 8.67 minutes. The concentrations measured over time in these experiments are reported in the tables below.

Table S3. Concentration data from kinetic experiments, RuPNP catalyst. Concentrations are in moles/liter.

RuPNP Experiment 1

$[Ru]_0$	$[epoxide]_0$	$P(H_2)$ (bar)	$[H_2]$
0.0005	0.5	20	0.09332

t (s)	[epoxide]	[2-TDOH]	[1-TDOH]
0	0.5	0	0
660	0.47767	0.01768	0.00061
1560	0.44228	0.05321	0.00062
2460	0.40355	0.08993	0.00093
3360	0.37873	0.11706	0.00129
4560	0.33968	0.15597	0.00149
5760	0.30767	0.18902	0.00181
6960	0.28023	0.21672	0.00189
8760	0.24493	0.25072	0.00203
10560	0.21661	0.27946	0.0022
12360	0.19465	0.30171	0.00231
14160	0.17256	0.32419	0.00241

RuPNP Experiment 3

$[Ru]_0$	$[epoxide]_0$	$P(H_2)$ (bar)	$[H_2]$
0.0015	0.5	20	0.09332

t (s)	[epoxide]	[2-TDOH]	[1-TDOH]
0	0.5	0	0
780	0.42523	0.06892	0.00077
1680	0.32544	0.17005	0.00164
2580	0.24169	0.25482	0.00216
3480	0.18473	0.31092	0.0025
4680	0.13272	0.36625	0.00284
5880	0.09674	0.40516	0.00312
7080	0.06999	0.42791	0.00323
8880	0.04689	0.45225	0.00333
10680	0.03179	0.46797	0.00337
12480	0.02297	0.47606	0.00338
14280	0.01676	0.48202	0.0034

RuPNP Experiment 2

$[Ru]_0$	$[epoxide]_0$	$P(H_2)$ (bar)	$[H_2]$
0.001	0.5	20	0.09332

t (s)	[epoxide]	[2-TDOH]	[1-TDOH]
0	0.5	0	0
720	0.44785	0.04586	0.00064
1620	0.3752	0.11961	0.0013
2520	0.30623	0.18901	0.00174
3420	0.25434	0.24003	0.00203
4620	0.20123	0.2942	0.00235
5820	0.16074	0.33665	0.00265
7020	0.12921	0.36761	0.00281
8820	0.09868	0.39918	0.00295
10620	0.07239	0.42431	0.00306
12420	0.05732	0.44019	0.00314
14220	0.0449	0.45245	0.0032

RuPNP Experiment 4

$[Ru]_0$	$[epoxide]_0$	$P(H_2)$ (bar)	$[H_2]$
0.001	0.5	10	0.04666

t (s)	[epoxide]	[2-TDOH]	[1-TDOH]
0	0.5	0	0
660	0.45494	0.04612	0.00072
1560	0.38009	0.12048	0.00116
2460	0.30516	0.19579	0.00163
3360	0.25453	0.24795	0.00203
4560	0.19502	0.30784	0.00231
5760	0.15364	0.35048	0.00257
6960	0.11835	0.38358	0.00285
8760	0.08492	0.41766	0.003
10560	0.06109	0.44335	0.0032
12360	0.04458	0.4609	0.0033
14160	0.03355	0.47012	0.00337

RuPNP Experiment 5

$[Ru]_0$	$[epoxide]_0$	$P(H_2)$ (bar)	$[H_2]$
0.001	0.5	30	0.13998

t (s)	[epoxide]	[2-TDOH]	[1-TDOH]
0	0.5	0	0
660	0.44421	0.04369	0.00067
1560	0.36583	0.12206	0.00114
2460	0.30465	0.19006	0.00173
3360	0.24437	0.24527	0.00186
4560	0.18875	0.30053	0.00219
5760	0.14797	0.34353	0.00258
6960	0.11686	0.37431	0.00275
8760	0.08456	0.40545	0.00286
10560	0.06148	0.42846	0.00306
12360	0.04617	0.44553	0.00317
14160	0.03495	0.45593	0.00324

RuPNP Experiment 6

$[Ru]_0$	$[epoxide]_0$	$P(H_2)$ (bar)	$[H_2]$
0.001	0.25	20	0.09332

t (s)	[epoxide]	[2-TDOH]	[1-TDOH]
0	0.25	0	0
600	0.22963	0.01747	0.00041
1500	0.19485	0.05313	0.00062
2400	0.16426	0.08457	0.00087
3300	0.13695	0.11189	0.0011
4500	0.11185	0.13761	0.00109
5700	0.08989	0.1596	0.00146
6900	0.07474	0.17531	0.00163
8700	0.05646	0.19313	0.00147
10500	0.04396	0.20606	0.00152
12300	0.03502	0.21522	0.0016
14100	0.02823	0.22279	0.0016

RuPNP Experiment 7

$[Ru]_0$	$[epoxide]_0$	$P(H_2)$ (bar)	$[H_2]$
0.001	0.75	20	0.09332

t (s)	[epoxide]	[2-TDOH]	[1-TDOH]
0	0.75	0	0
840	0.67115	0.06664	0.00088
1740	0.58909	0.1509	0.00174
2640	0.50306	0.23811	0.00231
3540	0.43494	0.30549	0.00267
4740	0.3617	0.3793	0.00311
5940	0.3081	0.43828	0.00354
7140	0.25671	0.48602	0.00372
8940	0.20468	0.54122	0.00402
10740	0.16502	0.58216	0.00425
12540	0.13454	0.60871	0.00438
14340	0.11248	0.62892	0.00449

Table S4. Concentration data from kinetic experiments, RuPNN catalyst. Concentrations are in moles/liter.

RuPNN Experiment 1

$[Ru]_0$	$[epoxide]_0$	$P(H_2)$ (bar)	$[H_2]$
0.0005	0.5	20	0.09332

t (s)	[epoxide]	[2-TDOH]	[1-TDOH]
0	0.5	0	0
660	0.46998	0.02912	0.00049
1560	0.44101	0.05673	0.00064
2460	0.40676	0.08814	0.00059
3360	0.37598	0.11624	0.00068
4560	0.33684	0.15221	0.0008
5760	0.30515	0.18068	0.00122
6960	0.27451	0.20907	0.00125
8760	0.24107	0.24015	0.00141
10560	0.2107	0.26567	0.00149
12360	0.18726	0.28576	0.00172
14160	0.16923	0.30036	0.00123

RuPNN Experiment 2

$[Ru]_0$	$[epoxide]_0$	$P(H_2)$ (bar)	$[H_2]$
0.001	0.5	20	0.09332

t (s)	[epoxide]	[2-TDOH]	[1-TDOH]
0	0.5	0	0
720	0.4384	0.0606	0.00057
1620	0.36889	0.12334	0.00099
2520	0.30626	0.18154	0.00121
3420	0.25638	0.22787	0.00146
4620	0.19954	0.27803	0.00155
5820	0.15984	0.31491	0.0017
7020	0.1261	0.34542	0.00184
8820	0.09135	0.37723	0.00193
10620	0.06639	0.40085	0.00205
12420	0.04768	0.41598	0.00166
14220	0.03635	0.42778	0.00175

RuPNN Experiment 3

$[Ru]_0$	$[epoxide]_0$	$P(H_2)$ (bar)	$[H_2]$
0.0015	0.5	20	0.09332

t (s)	[epoxide]	[2-TDOH]	[1-TDOH]
0	0.5	0	0
780	0.40487	0.09249	0.00079
1680	0.30944	0.18168	0.00143
2580	0.23413	0.25307	0.0018
3480	0.178	0.30384	0.00184
4680	0.12172	0.355	0.00196
5880	0.08573	0.38926	0.00214
7080	0.05917	0.41401	0.00227
8880	0.03547	0.43726	0.00167
10680	0.01999	0.45008	0.00177
12480	0.01315	0.45643	0.00176
14280	0.0086	0.45961	0.00204

RuPNN Experiment 4

$[Ru]_0$	$[epoxide]_0$	$P(H_2)$ (bar)	$[H_2]$
0.001	0.5	5	0.02333

t (s)	[epoxide]	[2-TDOH]	[1-TDOH]
0	0.5	0	0
480	0.47047	0.0218	0.00067
1380	0.44925	0.04568	0.00085
2280	0.41616	0.07116	0.00069
3180	0.38096	0.09676	0.00075
4380	0.345	0.12539	0.00101
5580	0.3071	0.15396	0.00109
6780	0.27383	0.17872	0.00113
8580	0.22946	0.20991	0.00117
10380	0.19542	0.23832	0.00135
12180	0.16498	0.25877	0.00121
13980	0.14037	0.27778	0.00143

RuPNN Experiment 5

$[Ru]_0$	$[epoxide]_0$	$P(H_2)$ (bar)	$[H_2]$
0.001	0.5	10	0.04666

t (s)	[epoxide]	[2-TDOH]	[1-TDOH]
0	0.5	0	0
600	0.45151	0.04608	0.00056
1500	0.41145	0.07958	0.00068
2400	0.36479	0.1207	0.00082
3300	0.32883	0.15622	0.00098
4500	0.27798	0.19491	0.00109
5700	0.23809	0.22918	0.00124
6900	0.20427	0.25924	0.00128
8700	0.162	0.29616	0.0014
10500	0.12697	0.32374	0.00141
12300	0.10039	0.34871	0.00149
14100	0.08028	0.3636	0.00155

RuPNN Experiment 6

$[Ru]_0$	$[epoxide]_0$	$P(H_2)$ (bar)	$[H_2]$
0.001	0.5	30	0.13998

t (s)	[epoxide]	[2-TDOH]	[1-TDOH]
0	0.5	0	0
600	0.42022	0.07153	0.0006
1500	0.34097	0.14755	0.00092
2400	0.27152	0.21302	0.00117
3300	0.21463	0.2666	0.00129
4500	0.16215	0.31727	0.00154
5700	0.11909	0.35583	0.00166
6900	0.08778	0.385	0.00182
8700	0.05729	0.41342	0.00192
10500	0.03786	0.43255	0.00188
12300	0.02608	0.44338	0.00191
14100	0.01743	0.45228	0.00205

RuPNN Experiment 7

$[Ru]_0$	$[epoxide]_0$	$P(H_2)$ (bar)	$[H_2]$
0.001	0.5	39	0.18197

t (s)	[epoxide]	[2-TDOH]	[1-TDOH]
0	0.5	0	0
540	0.41878	0.07558	0.00063
1440	0.33207	0.15926	0.00092
2340	0.25716	0.23189	0.00123
3240	0.1997	0.2888	0.00149
4440	0.13626	0.34578	0.00166
5640	0.09545	0.3851	0.00175
6840	0.06773	0.41213	0.0019
8640	0.04136	0.43859	0.00198
10440	0.02599	0.4521	0.00206
12240	0.01655	0.46184	0.00206
14040	0.01106	0.46914	0.00209

RuPNN Experiment 8

$[Ru]_0$	$[epoxide]_0$	$P(H_2)$ (bar)	$[H_2]$
0.001	0.25	20	0.09332

t (s)	[epoxide]	[2-TDOH]	[1-TDOH]
0	0.25	0	0
600	0.21483	0.03386	0.00026
1500	0.18207	0.06578	0.00064
2400	0.15108	0.09459	0.00066
3300	0.12575	0.11859	0.00079
4500	0.09801	0.14394	0.00085
5700	0.07772	0.16363	0.00095
6900	0.06071	0.17946	0.001
8700	0.04297	0.19599	0.0011
10500	0.03063	0.20719	0.00111
12300	0.02159	0.21466	0.00087
14100	0.0162	0.22058	0.00088

RuPNN Experiment 9

$[Ru]_0$	$[epoxide]_0$	$P(H_2)$ (bar)	$[H_2]$
0.001	0.75	20	0.09332

t (s)	[epoxide]	[2-TDOH]	[1-TDOH]
0	0.75	0	0
840	0.64751	0.09308	0.00085
1740	0.5583	0.17862	0.00158
2640	0.4781	0.2515	0.00168
3540	0.40784	0.31605	0.0019
4740	0.3273	0.38896	0.00216
5940	0.2655	0.44491	0.00237
7140	0.22109	0.49054	0.00256
8940	0.16018	0.5414	0.00266
10740	0.11712	0.57992	0.00225
12540	0.08897	0.6038	0.00242
14340	0.0697	0.62213	0.00249

Determination of the rate law and activation barrier for the RuPNP catalyst. As described in the main text, an application of the energetic span model to the MEP from computational analysis predicts the following rate law for the **RuPNP** catalyst system:

$$\mathrm{rate} = \mathrm{k[Ru][epoxide]}$$

In this equation, [Ru] represents the concentration of the TDI, which is **RuH^{PNP}**. Since no other intermediate is close in free energy, the concentration of **RuH^{PNP}** is approximately equal to the total ruthenium concentration in solution, and may be assumed to be constant over the time course of each reaction. The reaction is then expected to follow pseudo-first-order kinetics, according to this equation:

$$\mathrm{rate} = \mathrm{k_{obs}[epoxide]}, \text{ where } \mathrm{k_{obs}} = \mathrm{k[Ru]}$$

In integrated form, the rate law is:

$$\mathrm{ln[epoxide]} = -\mathrm{k_{obs}t} + \mathrm{ln[epoxide]_0}$$

The rate constant k can be calculated in each experiment as:

$$\mathrm{k} = \mathrm{k_{obs}/[Ru]}$$

Table S5 below shows this analysis for the seven kinetic experiments involving **Ru-MACHO-BH** as precatalyst.

Table S5. Determinate of the overall rate constant for the RuPNP system.

Experiment	$[\text{epoxide}]_0$ (M)	$P(H_2)$ (bar)	$[\text{Ru}]_0$ (M)	k_{obs} (s^{-1})	k ($s^{-1}\cdot M^{-1}$)
1	0.50	20	0.00050	0.0000762	0.1525
2	0.50	20	0.00100	0.0001731	0.1731
3	0.50	20	0.00150	0.0002443	0.1629
4	0.50	10	0.00100	0.0001956	0.1956
5	0.50	30	0.00100	0.0001914	0.1914
6	0.25	20	0.00100	0.0001583	0.1583
7	0.75	20	0.00100	0.0001352	0.1352
				mean	0.1670
				Std. dev	0.02148

Based on a rate constant of 0.167 ± 0.022 $s^{-1}\cdot M^{-1}$, application of the Eyring equation at the reaction temperature of 356.15 K gives an activation free energy of 22.24 ± 0.09 kcal/mol.

Determination of the rate law and activation barrier for the RuPNN catalyst. If the dihydride species **RuH^{PNN}** were the sole TDI, the same rate law described above for the **RuPNP** system would apply to the **RuPNN** system. If the alkoxide intermediate **RuO^iPr^{PNN}** is close enough in free energy to occupy a significant fraction of the ruthenium speciation under catalytic conditions, a more complicated rate law will apply, as determined by the sequence shown in Scheme 2 in the main text. In the following derivation, we have assumed that the hydrogenolysis product 2-tetradecanol will interact with the ruthenium species similarly to the 2-propanol solvent. Since the solvent at 13.1 M is always at a much higher concentration than the product, we have ignored the 2-tetradecanol product in the kinetic analysis.

The two-step sequence in Scheme 2 can be written in linear form as:

$$1)\ \mathrm{RuO^iPr} + \mathrm{H_2} \underset{k_{-1}}{\overset{k_1}{\rightleftharpoons}} \mathrm{RuH} + {}^{\mathrm{i}}\mathrm{PrOH}$$

$$2)\ \mathrm{RuH} + \mathrm{epoxide} \overset{k_2}{\rightarrow} \mathrm{RuO^iPr}$$

If **RuO^iPr** and **RuH** are the dominant ruthenium species present, we can represent their sum as Ru_{total}:

$$[\mathrm{Ru_{total}}] = [\mathrm{RuH}] + [\mathrm{RuO^iPr}]$$

We can apply the steady-state approximation to the **RuH** intermediate:

$$\frac{d[\mathrm{RuH}]}{dt} = 0 = k_1[\mathrm{RuO^iPr}]\,[\mathrm{H_2}] - k_{-1}[\mathrm{RuH}][{}^{i}\mathrm{PrOH}] - k_2[\mathrm{RuH}][\mathrm{epoxide}]$$

Substituting $[\mathrm{Ru_{total}}]$ - [RuH] for [RuO^{i}Pr] gives:

$$0 = k_1([\mathrm{Ru_{total}}] - [\mathrm{RuH}])[\mathrm{H_2}] - k_{-1}[\mathrm{RuH}][^i\mathrm{PrOH}] - k_2[\mathrm{RuH}][\mathrm{epoxide}]$$

Solving for [RuH] gives:

$$[\mathrm{RuH}] = \frac{k_1[\mathrm{Ru_{total}}][\mathrm{H_2}]}{k_1[\mathrm{H_2}] + k_{-1}[^i\mathrm{PrOH}] + k_2[\mathrm{epoxide}]}$$

From reaction 2 above, the rate of product formation (or epoxide consumption) is:

$$\frac{dP}{dt} = k_2[\mathrm{RuH}][\mathrm{epoxide}]$$

Substituting in the steady-state concentration of RuH gives:

$$\frac{dP}{dt} = \frac{k_1 k_2[\mathrm{Ru_{total}}][\mathrm{H_2}][\mathrm{epoxide}]}{k_1[\mathrm{H_2}] + k_{-1}[^i\mathrm{PrOH}] + k_2[\mathrm{epoxide}]}$$

Here, we can make a simplifying assumption that $k_2[\mathrm{epoxide}]$ is negligible in the denominator. If $k_2[\mathrm{epoxide}]$ were significant, saturation behavior in [epoxide] would be observed at high epoxide concentrations. This assumption is also consistent with the energy barriers predicted by DFT. The barrier from **RuH$^{\mathbf{PNN}}$** to **h-TS$^{\mathbf{PNN}}$** (corresponding to k_2) is 25.1 kcal/mol, while the barrier from **RuH$^{\mathbf{PNN}}$** backwards to **e-TS$^{\mathbf{PNN}}$** (corresponding to k_{-1}) is 19.1 kcal/mol. This assumption gives the rate law:

$$\frac{dP}{dt} = \frac{k_2[\mathrm{Ru_{total}}][\mathrm{H_2}][\mathrm{epoxide}]}{[\mathrm{H_2}] + \frac{k_{-1}}{k_1}[^i\mathrm{PrOH}]}$$

At this point, reaction 1 is a fast pre-equilibrium and reaction 2 is rate-determining. We can replace $\frac{k_{-1}}{k_1}$ with the reciprocal of the equilibrium constant K_1 for reaction 1:

$$\frac{dP}{dt} = \frac{k_2[\mathrm{Ru_{total}}][\mathrm{H_2}][\mathrm{epoxide}]}{[\mathrm{H_2}] + \frac{1}{K_1}[^i\mathrm{PrOH}]}$$

In a kinetic experiment where epoxide is consumed while $[\mathrm{Ru_{total}}]$ and $[\mathrm{H_2}]$ are constant, the epoxide is the only variable:

$$\frac{dP}{dt} = \frac{k_2[Ru_{total}][H_2]}{[H_2] + \frac{1}{K_1}[^iPrOH]}[epoxide]$$

So the pseudo-first-order rate law applies:

$$\ln[epoxide] = -k_{obs}t + \ln[epoxide]_0$$

where:

$$k_{obs} = \frac{k_2[Ru_{total}][H_2]}{[H_2] + \frac{1}{K_1}[^iPrOH]}$$

To analyze all 9 kinetics experiments together, we can incorporate $[Ru_{total}]$ into k_{obs}:

$$\frac{k_{obs}}{[Ru_{total}]} = \frac{k_2[H_2]}{[H_2] + \frac{1}{K_1}[^iPrOH]}$$

We can then plot $\frac{k_{obs}}{[Ru_{total}]}$ vs $[H_2]$ and determine k_2 and K_1 through a least-squares fit. Table S6 below shows the determination of $\frac{k_{obs}}{[Ru_{total}]}$ and $[H_2]$, calculated using the Henry's law constant of 0.004666 M/bar for hydrogen in 2-propanol at 356.15 K.[12]

Table S6. Kinetics calculations for the **RuPNN** system.

Experiment	$[epoxide]_0$ (M)	$P(H_2)$ (bar)	$[H_2]$ (M)	$[Ru]_{total}$ (M)	k_{obs} (s^{-1})	$k_{obs}/[Ru]_{tot}$ ($s^{-1}\cdot M^{-1}$)
1	0.50	20	0.09332	0.00050	0.0000783	0.1566
2	0.50	20	0.09332	0.00100	0.0001871	0.1871
3	0.50	20	0.09332	0.00150	0.0002911	0.1940
4	0.50	5	0.02333	0.00100	0.0000914	0.0914
5	0.50	10	0.04666	0.00100	0.0001296	0.1296
6	0.50	30	0.13998	0.00100	0.0002391	0.2391
7	0.50	39	0.18197	0.00100	0.0002759	0.2759
8	0.25	20	0.09332	0.00100	0.0002746	0.2746
9	0.75	20	0.09332	0.00100	0.0002735	0.2735

Figure S5 below shows the plot of $\frac{k_{obs}}{[Ru_{total}]}$ vs $[H_2]$. The gray line represents a least-squares fit, where K_1 = 96.5 ± 35.1 and k_2 = 0.461 ± 0.093 $M^{-1} \cdot s^{-1}$.

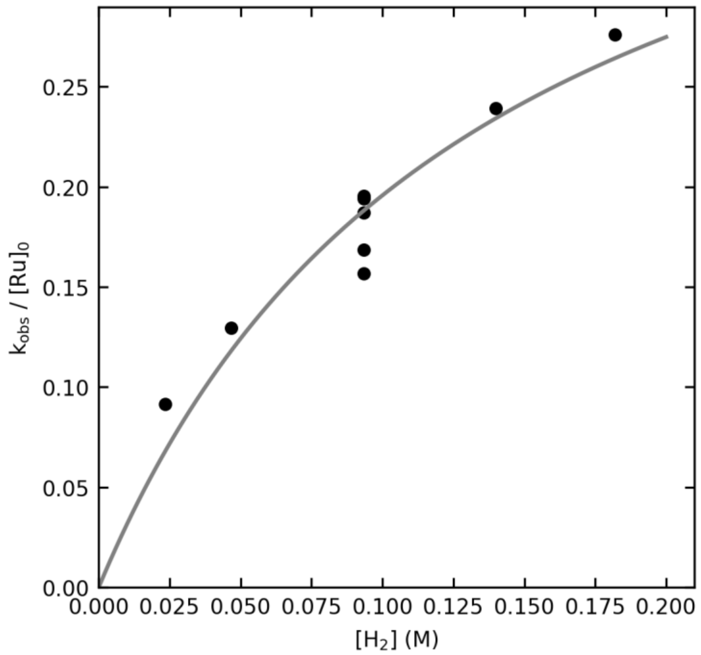

Figure S5. Determination of K_1 and k_2 for the **RuPNN** system.

Using the equation $\Delta G° = -RT \ln K$ at the reaction temperature of 356.15 K, we find that $\Delta G°$ for reaction 1 is -3.32 ± 0.26 kcal/mol. Using the Eyring equation, we find that $\Delta G^{‡}$ for reaction 2 is 21.52 ± 0.14 kcal/mol.

References

(1) (a) He, T.; Buttner, J. C.; Reynolds, E. F.; Pham, J.; Malek, J. C.; Keith, J. M.; Chianese, A. R. Dehydroalkylative Activation of CNN- and PNN-Pincer Ruthenium Catalysts for Ester Hydrogenation. *J. Am. Chem. Soc.* **2019,** *141*, 17404-17413. (b) Pham, J.; Jarczyk, C. E.; Reynolds, E. F.; Kelly, S. E.; Kim, T.; He, T.; Keith, J. M.; Chianese, A. R. The Key Role of the Latent N–H Group in Milstein's Catalyst for Ester Hydrogenation. *Chem. Sci.* **2021,** *12*, 8477-8492.

(2) Frisch, M. J.; Trucks, G. W.; Schlegel, H. B.; Scuseria, G. E.; Robb, M. A.; Cheeseman, J. R.; Scalmani, G.; Barone, V.; Petersson, G. A.; Nakatsuji, H.; Li, X.; Caricato, M.; Marenich, A. V.; Bloino, J.; Janesko, B. G.; Gomperts, R.; Mennucci, B.; Hratchian, H. P.; Ortiz, J. V.; Izmaylov, A. F.; Sonnenberg, J. L.; Williams; Ding, F.; Lipparini, F.; Egidi, F.; Goings, J.; Peng, B.; Petrone, A.; Henderson, T.; Ranasinghe, D.; Zakrzewski, V. G.; Gao, J.; Rega, N.; Zheng, G.; Liang, W.; Hada, M.; Ehara, M.; Toyota, K.; Fukuda, R.; Hasegawa, J.; Ishida, M.; Nakajima, T.; Honda, Y.; Kitao, O.; Nakai, H.; Vreven, T.; Throssell, K.; Montgomery Jr., J. A.; Peralta, J. E.; Ogliaro, F.; Bearpark, M. J.; Heyd, J. J.; Brothers, E. N.; Kudin, K. N.; Staroverov, V. N.; Keith, T. A.; Kobayashi, R.; Normand, J.; Raghavachari, K.; Rendell, A. P.; Burant, J. C.; Iyengar, S. S.; Tomasi, J.; Cossi, M.; Millam, J. M.; Klene, M.; Adamo, C.; Cammi, R.; Ochterski, J. W.; Martin, R. L.; Morokuma, K.; Farkas, O.; Foresman, J. B.; Fox, D. J. *Gaussian 16 Rev. B.01*, Wallingford, CT, 2016.

(3) Chai, J.-D.; Head-Gordon, M. Long-Range Corrected Hybrid Density Functionals with Damped Atom–Atom Dispersion Corrections. *Phys. Chem. Chem. Phys.* **2008,** *10*, 6615-6620.

(4) Weigend, F.; Ahlrichs, R. Balanced Basis Sets of Split Valence, Triple Zeta Valence and Quadruple Zeta Valence Quality for H to Rn: Design and Assessment of Accuracy. *Phys. Chem. Chem. Phys* **2005,** *7*, 3297-3305.

(5) Marenich, A. V.; Cramer, C. J.; Truhlar, D. G. Universal Solvation Model Based on Solute Electron Density and on a Continuum Model of the Solvent Defined by the Bulk Dielectric Constant and Atomic Surface Tensions. *J. Phys. Chem. B* **2009,** *113*, 6378-6396.

(6) Dub, P. A.; Gordon, J. C. The Mechanism of Enantioselective Ketone Reduction with Noyori and Noyori-Ikariya Bifunctional Catalysts. *Dalton Trans.* **2016,** *45*, 6756-6781.

(7) Cramer, C. J., In *Essentials of Computational Chemistry*, 2nd ed.; Wiley: Chichester, UK, 2004; 378-379.

(8) Becke, A. D. Density-Functional Thermochemistry. III. The Role of Exact Exchange. *J. Chem. Phys.* **1993,** *98*, 5648-5652.

(9) Grimme, S.; Antony, J.; Ehrlich, S.; Krieg, H. A Consistent and Accurate Ab Initio Parametrization of Density Functional Dispersion Correction (DFT-D) for the 94 Elements H-Pu. *J. Chem. Phys.* **2010,** *132*, 154104.

(10) Zhao, Y.; Truhlar, D. G. A New Local Density Functional for Main-Group Thermochemistry, Transition Metal Bonding, Thermochemical Kinetics, and Noncovalent Interactions. *J. Chem. Phys.* **2006,** *125*, 194101.

(11) Yu, H. S.; He, X.; Li, S. L.; Truhlar, D. G. MN15: A Kohn-Sham Global-Hybrid Exchange-Correlation Density Functional with Broad Accuracy for Multi-Reference and Single-Reference Systems and Noncovalent Interactions. *Chem Sci* **2016,** *7*, 5032-5051.

(12) Brunner, E. Solubility of Hydrogen in Alcohols. *Ber. Uunsenges. Phys. Chem.* **1979,** *83*, 715-721.
